# Impacts of climate change on mangrove subsistence fisheries: a global review

**DOI:** 10.1007/s42995-024-00231-3

**Published:** 2024-06-05

**Authors:** Roann P. Alberto, Judith A. Teano, Annie Melinda Paz-Alberto, Mark Anthony B. Tangonan, Hazel Jade E. Villamar, Sarah Clement, David J. S. Montagnes, Andrew P. Morse

**Affiliations:** 1 College of Business Administration and Accountancy, Central Luzon State University, 3120 Science City of Munoz, Nueva Ecija Philippines; 2 Environmental Economics, College of Business Administration and Accountancy, Central Luzon State University, 3120 Science City of Munoz, Nueva Ecija Philippines; 3 Ecology and Environmental Management, Institute for Climate Change and Environmental Management, Retired University Professor, Central Luzon State University, 3120 Science City of Munoz, Nueva Ecija Philippines; 4 Accountancy and Finance, College of Business Administration and Accountancy, Central Luzon State University, 3120 Science City of Munoz, Nueva Ecija Philippines; 5 Environmental Policy, Australian National University, Canberra, ACT 2601 Australia; 6 Department of Evolution, Ecology & Behaviour, University of Liverpool, BioSciences Building, Crown Street, Liverpool, L69 7ZB UK; 7 School of Environmental Sciences, Roxby Building, University of Liverpool, Liverpool, L69 7ZT UK

**Keywords:** Climate change, Mangrove stocks, Flooding, Social impact, Subsistence fishers, Typhoons

## Abstract

Climate change will impact coastal ecosystems, threatening subsistence fisheries including those in mangrove forests. Despite their global contributions and roles in nutrition and cultural identity, mangrove subsistence fisheries are poorly studied. Here, we offer a foundation for improving the management of mangrove subsistence fisheries to deal with the impending effects of climate change. This multidisciplinary review—drawing on organismal biology, ecology, fisheries, and social science—focuses on the climate impacts relevant to mangrove ecosystems: heat waves, low-category, and high-category typhoons. First, we provide an overview of the mangroves, their harvestable stocks (fish, crustaceans, molluscs), and the fishers, offering an understanding of how they may be affected by relevant environmental variables; i.e., shifts in temperature, salinity, oxygen, flooding, and sediments. Then, we examine the potential effects of climate change on mangrove stocks and fishers, indicating the scope of impending changes. By combining the above information, we develop a simple model that forecasts the number of “fishing-days” lost by fishers due to climate change over the next decade (between 11 and 21 days will be lost per year per fisher). This indicates which aspects of climate change will have the greatest impacts on stocks and fishers. We found that high-category typhoons had more impacts than heat waves, which in turn had a greater impact than low-category typhoons). Finally, recognising gaps in our knowledge and understanding, we offer recommendations for approaches for future work to improve our predictions.

**Supplementary Information:**

The online version contains supplementary material available at 10.1007/s42995-024-00231-3.

## Introduction

Climate change poses risks to coastal ecosystems (He and Silliman 2019), affecting subsistence fishers in rural areas (Montejo-Damian et al. 2022). These fisheries, contributing 50% to global marine catches and ~ 70% to human consumption, play critical roles in nutrition and economics; furthermore, they foster community cohesion through a shared sense of identity and informal economies (FAO 2015; Khakzad and Griffith 2016). Despite their importance, subsistence fisheries receive inadequate attention, particularly in the context of climate change (Islam and Berkes 2016). Here, we review the literature on one globally dispersed set of subsistence fisheries that has received little attention and will be undoubtedly affected by climate change—those in mangrove forests. Specifically, to address immediate issues, we focus mainly on impacts over the next decade. Our analysis is multidisciplinary, drawing on the fields of organismal biology, ecology, fisheries, climate science, and social science. Besides our proximal aim, which is to assess the impacts of climate change on mangrove subsistence fishers, we see our efforts as a “proof of concept,” for applying multidisciplinary approaches to uncover priority actions for mitigating climate change damages.

To ensure an understanding of this complex topic, firstly we provide an overview of the mangroves, their harvestable stocks, and their fishers, using data directly from mangrove studies but also augmenting these with other estuarine data (*Mangrove forests, their subsistence fisheries, and fishers*). Then, we examine the potential effects of climate change on mangrove stocks and fishers, specifically indicating which aspects of climate change will have the greatest impacts and which are of less concern *(Climate impacts on the mangrove subsistence stocks and fishers*). Next, we apply our findings by combining the above information to develop a model that forecasts the “number of days lost” (as a currency of impact) by fishers due to climate change over the next decade; this allowed us to concentrate on first-order effects (*An estimate of fishing-days lost due to climate impacts: a case study*). The model, a synthesis of extensive research, significantly advances our understanding of the multifaceted impacts of climate change on mangrove subsistence fishers. By offering an empirical method to quantify direct effects, it provides a detailed view of their challenges and proves invaluable in translating research into practical climate impact insights. Finally, recognising gaps in our knowledge and understanding, we offer recommendations for approaches now needed to ensure that mangrove subsistence fishers are prepared for the inevitable changes on the horizon (*Outlook*). This guidance may ultimately apply to stakeholders and policymakers. However, our main aim is, by making this first critical multidisciplinary evaluation, we can offer guidance for researchers on how they might conduct much needed studies to improve our understanding.

## Mangrove forests, their subsistence fisheries, and fishers

In this section, we introduce the relevant biota in the mangrove ecosystem: the mangrove forests, the main stocks, and the fishers. By providing this overview of their basic biology, we indicate, in general, how they may or may not be vulnerable to environmental change. Such a fundamental understanding of the “key players” is essential prior to appreciating how climate change will affect the mangrove subsistence fishers and their stocks. Then, we summarise our literature search on the ecophysiological limitations of key players in a later section: *Climate impacts on the mangrove subsistence stocks and fishers*.

### Mangrove forests

Mangrove trees (5–50 m high) occur in tropical, estuarine waters with branched roots anchored in loose sediments (Kathiresan and Bingham 2001). As the trees can withstand salinity changes, extreme waves, strong winds, high temperatures and muddy anaerobic soils, they are resilient to some of the effects of climate change (Alongi 2008). Moreover, mangroves grow rapidly, thriving where water sediment loads are 10–300 g/L, where shores accrete sediment at 0.5 cm/y to 1 cm/y, and where erosion occurs; these attributes should allow them to survive the predicted climate-change induced changes in sea level (Ellison 1999). However, as indicated below, some aspects of climate change will affect them.

Of relevance to fishing, the submerged mangrove roots directly and indirectly provide habitats for harvestable stocks and nurseries for stocks where the adults live elsewhere, such as marketable pelagic fish (Bimrah et al. 2022). In addition, these forests bolster coastal ecosystems and subsistence fishing areas by mitigating the effects of waves and storm surges on both the biotic community and nearby fishers (Alongi 2015; Bimrah et al. 2022).

Mangroves are essential to over 4.1 million subsistence fishers worldwide, providing nearly 80 million tonnes of fish each year, valued at more than US $50,000 per hectare, and sustaining the economies of coastal communities in over 100 countries and territories (Hutchinson et al. 2014; zu Ermgassen et al. 2020). The fisheries, however, cannot be viewed as a single entity in terms of the effects of climate change, because the ecosystem hosts several functionally distinct, harvestable stocks (Table 1). In the following sections we examine these stocks and the resident fishers before addressing how climate change may affect them.

**Table 1:** Mangrove stocks and their associated fishing methods

Fishery group	Fishing location	Common species collected in mangroves or mangrove-associated areas*	Fishing technologies/gear	Sector	Consumption	Study location
Finfish	Mangrove^1,6,12,17^	Mullet (Mugilidae)^1,2,6,10^, Rabbitfish *(*Siganidae) & Spinefoot ^1,2,6,10^ (*Siganus lineatus; Siganus guttatus; Siganus vermicuatus)*^1,2,6,10^	Cast and gill nets^6,12^	Subsistence^,6,14^	Fishers and their family^4,6,12,14,20^	Australia,^11^ Bangladesh,^7,13,15,18^ Guinea,^3^ Indonesia,^18,19^ Malaysia,^17^ Philippines,^13,18^
	Mangrove, open estuary, offshore^1,6^	Sea bream (Sparidae*)*,^1,2,6,10^, Gobies (Gobiidae),^1,2,6,10^, Striped sea bass (*Morone saxatilis*)^1,2,6,10^, Eel (Anguillidae and Muraenidae)^1,2,6,10^	Trawls, nets, hook and lines^6,12^	Subsistence, commercial^6,14^	Sold and traded in markets^4,6,14,20^	Australia,^11^ Bangladesh,^7,13,15,18^ Guinea,^3^ Indonesia,^18,19^ Malaysia,^17^ Philippines,^13,18^
	Mangrove, open estuary, offshore^1,6^	Mangrove red snapper (*Lutjanus grisius*; *Lutjanus argentimaculatus)*^1,2,6,10^, Barramundi (*Lates calcarifer)*^1,2,6,10^	Trawls, nets, hook and lines, spear, lures^6,12^	Subsistence, commercial, recreational^6,14^	Fishers and family, but mostly sold in markets^4,6,14,20^	Australia,^11^ Bangladesh,^7,13,18^ Guinea,^3^ Indonesia,^18,19^ Malaysia,^17^ Philippines,^13,18^
Crustaceans	Mangrove^,1,6^	Mud crab (*Scylla* spp.*)*^2,6,10,11,19^	Hand collection, weir traps and pots^6,12^	Subsistence^6,14^	Fisher and their family^6,14,20^	Australia,^11^ Bangladesh,^7,13,18^ Indonesia,^18,19^ Philippines,^13,15,18^
	Mangrove, open estuary, offshore^1,5,6,17^	Prawn (*Penaeus* spp.)^2,6,17,19^	Nets, Trawls, Rectangular boards^6,12^	Subsistence, commercial^6,14^	Sold in markets and traded^6,14^	Australia,^11^ Bangladesh,^7,13,18^ Indonesia,^18,19^ Malaysia,^17^ Mozambique,^16,18^ Philippines,^13,18^ Vietnam,^5^
Mollusc	Mangrove^1,6^	Oysters (*Crassostrea* spp.)^6,8,9,10 19^	Hand collection by cutting the mangrove roots (axe & picks)^6,12^	Subsistence, small-scale^6,14^	Fishers and their family^,6,14,20^	Africa,^9^ America,^9^ Australia,^11^ Bangladesh,^7,8,13,18^ Indonesia,^18,19^ Philippines,^13,17,18^

As there are few studies on mangrove fish communities, the organisms listed are those caught most often in mangrove areas, as shown by the citations*

^*^Data are from ^1^Blaber (2007), ^2^Carrasquilla-Henao et al. (2019), ^3^Cravo et al. (2021), ^4^de Boer et al. (2020), ^5^de Graaf and Xuan (1998), ^6^Hutchinson et al. (2014), ^7^Islam and Haque (2004), ^8^Jana et al. (2013), ^9^Lapègue et al. (2002), ^10^Manson et al. (2005), ^11^Meynecke et al. (2007), ^12^Monteclaro et al. (2017), ^13^Mozumber et al. (2018), ^14^Palomares and Pauly (2019), ^15^Primavera (2017), ^16^Rönnbäck et al. (2002), ^17^Sasekumar et al. (1992), ^18^Seary (2019), ^19^Seary et al. (2021), ^20^Tacon and Metian (2017)

### Finfish

Globally, finfish (henceforth fish) provide nutritional and economic benefits to subsistence fishers, because they are rich in protein and essential micronutrients (FAO 2020). Fish are widely available, less expensive than most other protein sources, and can be caught passively or actively for personal consumption or market sale (Table 1). The mangrove ecosystem supports a diverse range of fish caught by subsistence fishers (Table 1); understanding their functional diversity and how environmental factors may affect them is needed to evaluate the effects of climate change.

Fish, being ectothermic, adjust to ambient water temperature and generally prefer oxygen-rich, sediment-free environments for efficient feedirefereng, predator evasion, and respiration (Helfman et al. 2009; Kjelland et al. 2015). Mangrove species are also typically euryhaline and eurythermal due to fluctuating temperatures and salinities in their estuarine habitats (Illari et al. 2022). Still, optimal conditions (temperature, salinity, oxygen) dictate the spatial and temporal distribution and success of populations, so substantial shifts in the environment may be detrimental (Madeira et al. 2012; Pankhurst and Munday 2011).

Some fish are resident spawners in mangrove estuaries, whereas others, either anadromous or catadromous, breed in marine or freshwater habitats, respectively (Potter et al. 2013). Given their migratory patterns (Rulifson 1989), they may also return to their habitats when displaced; i.e., many possess homing behaviours (White and Brown 2013). When fish spawn, their eggs may float (pelagic) with the currents or sink (demersal) and attach to structures such as mangrove roots (Kunz 2004). These eggs develop into juveniles that either migrate to the mangrove forests, aided by ocean currents, or seek refuge immediately if they are permanent residents (Llopiz et al. 2014). The diversity of these breeding behaviours means that assessing the climate impacts on the survival and recruitment of mangrove fish is complex and often stock-specific. Below (*Climate impacts on the mangrove subsistence stocks and fishers*), we do not attempt to address all stocks but rather recognise the wide breadth of behaviours and focus on the environmental pressures that might affect them, using at times non-mangrove (but tropical-estuarine) species as surrogates to indicate trends.

### Crustaceans

Mud crabs, *Scylla* spp., and prawns, *Penaeus* spp., which are rich in protein and micronutrients, are by far the crustaceans most caught by mangrove subsistence fishers (Hutchinson et al. 2014; Susanto 2021). Although both stocks are eurythermal and euryhaline as adults (Motoh 1985; Pati et al. 2023) and can move to avoid local fluctuations (e.g., in oxygen, Zheng et al. 2021), changes within the estuary will affect them, as discussed in more detail below in *Climate impacts on the mangrove subsistence stocks and fishers*. As with fish, here we simply introduce these stocks.

Mud crabs inhabit burrows among mangrove roots and adjacent sediments (Alberts-Hubatsch et al. 2015). In addition, they spend some time on land, allowing them to escape environmental changes in the water (Pati et al. 2023). Crabs require little skill or equipment to collect, being typically caught by non-stationary gear (Table 1). These crabs share some characteristics with fish: both are ectothermic, catadromous, visual-feeders and rely on gills to respire (Alberts-Hubatsch et al. 2015). However, unlike fish, they are only semi-mobile, and do not exhibit homing behaviours (Alberts-Hubatsch et al. 2015). Mud crabs store their sperm and eggs until a suitable marine location to spawn is found, where they release fertilised eggs that develop and disperse by prevailing currents to new locations (Hewitt et al. 2023). As they mature, mud crabs return to the mangrove forests and inhabit brackish waters (Pati et al. 2023).

Penaeid prawns are favoured by subsistence fishers for their rapid growth and high nutritional content (Gillett 2008). Much like fish, they are motile and are caught with a variety of stationary and non-stationary gear (Table 1). In addition, they share traits with mud crabs, being ectothermic, and relying on gills for respiration (Henry et al. 2012). Moreover, they do not exhibit homing behaviours; rather they spawn at sea where their eggs sink and then become planktonic larvae (Motoh 1985). These larvae then rely on ocean currents to settle in inshore and estuarine waters where they spend their juvenile to adult life before emigrating offshore to complete the cycle (Vance et al. 2002).

### Molluscs (i.e., oysters)

Although other molluscs may occasionally be harvested, oysters (*Crassostrea* spp.) are the primary stocks harvested by mangrove subsistence fishers (Hutchinson et al. 2014). Oysters, like fish and crustaceans, have high nutritional value and are abundant along the coast (Negara et al. 2022), and as they are sessile, living on roots just below the surface, they are relatively easy to harvest, needing little gear to collect (Table 1).

As filter feeders, oysters require clear water that supports growth of their phytoplankton prey (Dame 2012). Like fish and crustaceans, they are ectothermic, euryhaline, eurythermal, and need optimal conditions for them to thrive (Gosling 2004). While they are capable of enduring long periods in freshwater, very low oxygen (0%–1% saturation) and salinity (< 3) may impact their recruitment and growth (Mclachla and Erasmus 1974; Rivera-Ingraham and Lignot 2017). However, if conditions (e.g., salinity, oxygen, sediments) become poor for short periods (< 24 h), oysters close their shells and escape environmental change (Goncalves et al. 2018).

Mangrove-associated oysters live their entire lives within these estuaries. The sessile adults produce gametes that are dispersed into the estuarine waters (Dame 2012), where the fertilized eggs settle and hatch into the planktonic stage, veliger larvae; juveniles then develop and attach to mangrove roots (Gosling 2004). Therefore, unlike finfish and crustaceans, oysters will be affected only by changes within the estuary, as detailed in *Climate impacts on the mangrove subsistence stocks and fishers*.

### Subsistence fishers, their homes, health, and technologies

**Fig. 1:**
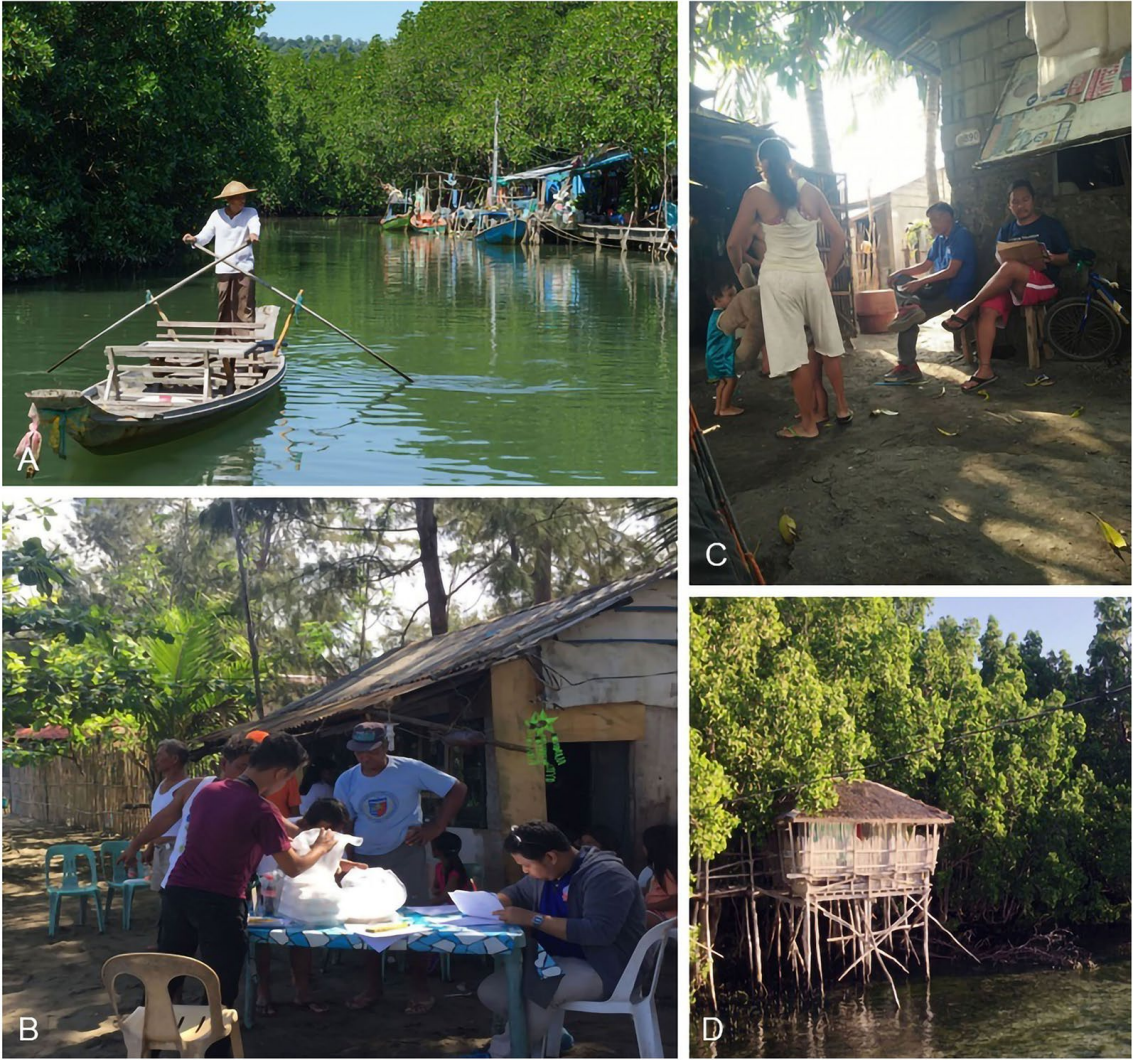
Examples of mangrove subsistence fishers and their habitats, from the Philippines. **A** Area within the mangroves where fishing occurs. **B**, **C** Houses close together, with narrow roads in a coastal area. **D** Shaded house within a mangrove forest, typically made of thin straws, bamboo, and dried coconut leaves

Data on mangrove subsistence fishers are limited; however, extrapolation from knowledge of human physiology and reports from regions similar to mangrove forests, suggests that climate change will affect their housing, health, and fishing. Subsistence fishers are mostly poor, small-scale processors, and traders (Arthur et al. 2021). They live close to mangrove waters, usually in densely populated coastal areas with narrow roads, poorly shaded houses, and informal settlements (Fig. 1). Suboptimal housing conditions impact on their health, particularly in tropical regions where high temperatures (> 36 °C) and extreme weather events increase risks of stress, diseases, and mortality (Mora et al. 2022; Woodhead et al. 2018).

Aside from the above challenges, the living conditions of subsistence fishers limit their access to schools and other educational resources, resulting in most being illiterate (e.g., Bhuyan and Islam 2016—Bangladesh, Branch et al. 2002—South Africa, Khatua 2022—India, Kinseng et al. 2019—Indonesia, Knudsen 2016—Philippines). Consequently, fishers tend to rely on traditional knowledge and beliefs (Adjei and Sika-Bright 2019; de Sousa et al. 2022) and local ecological knowledge (Hiwasaki et al. 2014). This illiteracy will affect many aspects of their lives, from their fishing schedules to their low-technology “artisanal” gear (Wekke and Cahaya 2015).

**Fig. 2:**
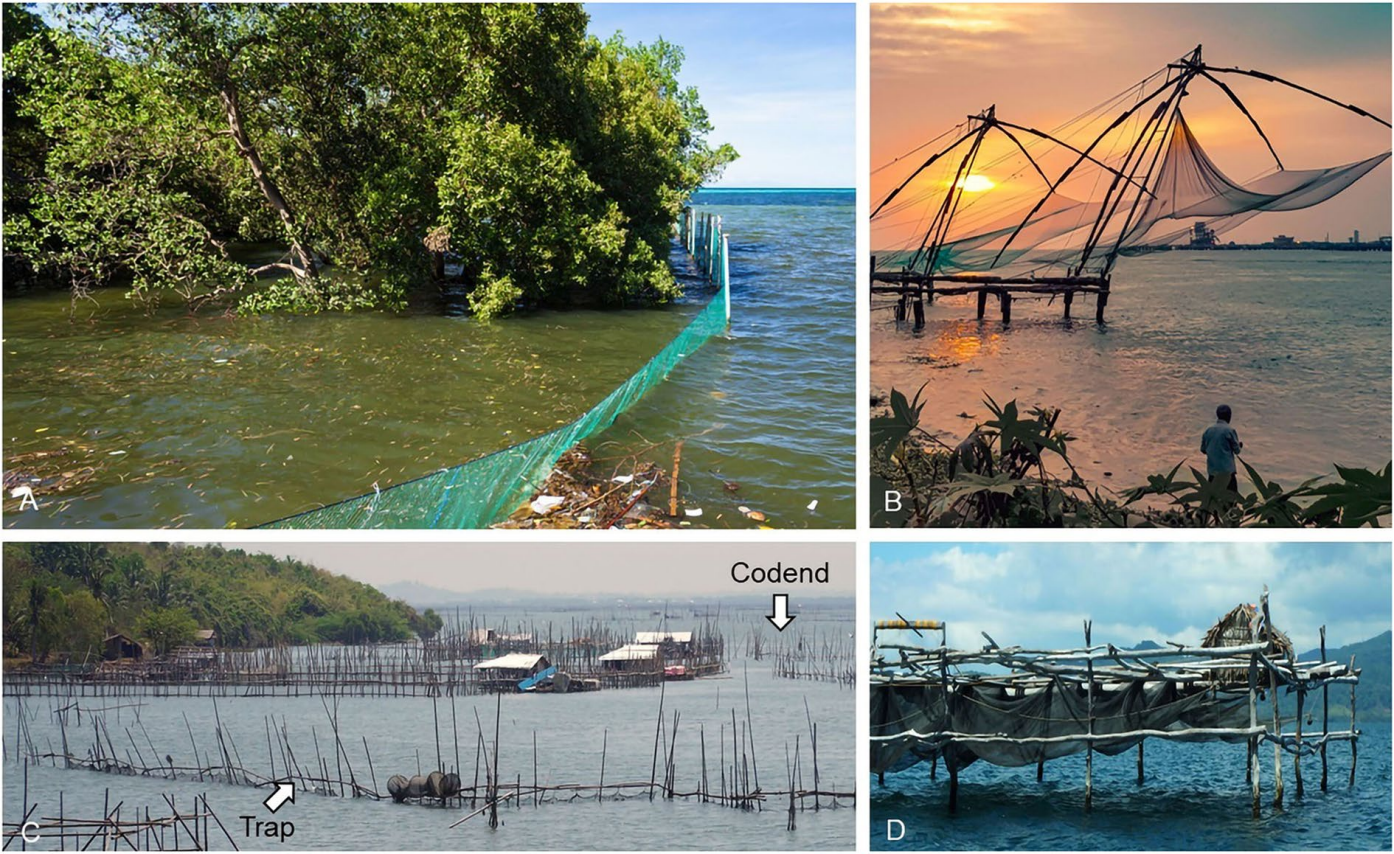
Stationary or passive gear often used in tropical regions. **A** Barrier nets to catch daily migrating fish in mangrove areas. **B** Lift nets attract fish over the submerged net, which is hoisted by an improvised pulley system. **C** Tidal trap with wings that guide prawns and fish to the codend for capture. **D** Filter nets that form a conical bag at the end and trap fish (for details on this gear see He et al. 2021; Monteclaro et al. 2017; Sultana and Islam 2016)

**Fig. 3:**
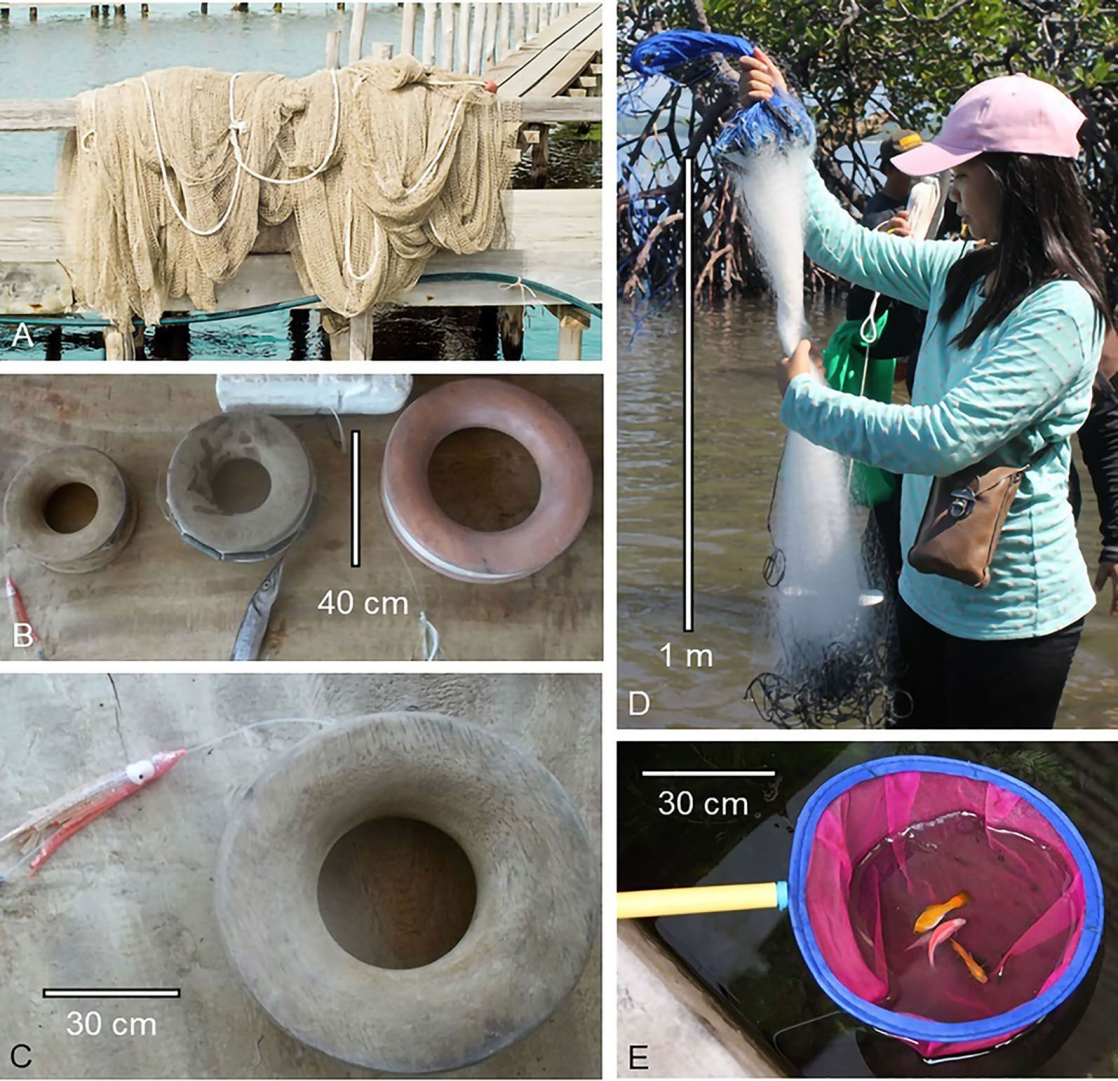
Non-stationary or active gear in tropical regions. **A** Seine used to sieve the water column to catch fish. **B** Kawil baited and coiled on a wooden handle, catch fish and prawns. **C** Crab pot lures with bait and closes to trap crabs inside. **D** Gill nets set near the surface or at the bottom will trap fish. **E** Scoop nets are conical and hand-held to capture fish in shallow waters (for details on this gear see He et al. 2021; Monteclaro et al. 2017)

Often, subsistence fishers use traditional fishing gear and methods (Quinn 2009). Although traditional fishing may vary by region, here we detail those that are commonly used in the Philippines as they should be indicative of other areas (He et al. 2021; Monteclaro et al. 2017). There are two types of fishing gear used—stationary (Fig. 2) and non-stationary (Fig. 3)—both of which are typically handmade from inexpensive and easily accessible materials, such as bamboo, plywood, nylon nets, pots, and sticks (Monteclaro et al. 2017). By its very nature such simple gear is fragile and subject to destruction by aspects of climate change (i.e., sunlight, winds, flooding), but it is also inexpensive and relatively easy to replace, assuming the fishers have funds and resources to do so (Monteclaro et al. 2017).

## Climate impacts on the mangrove subsistence stocks and fishers

**Fig. 4:**
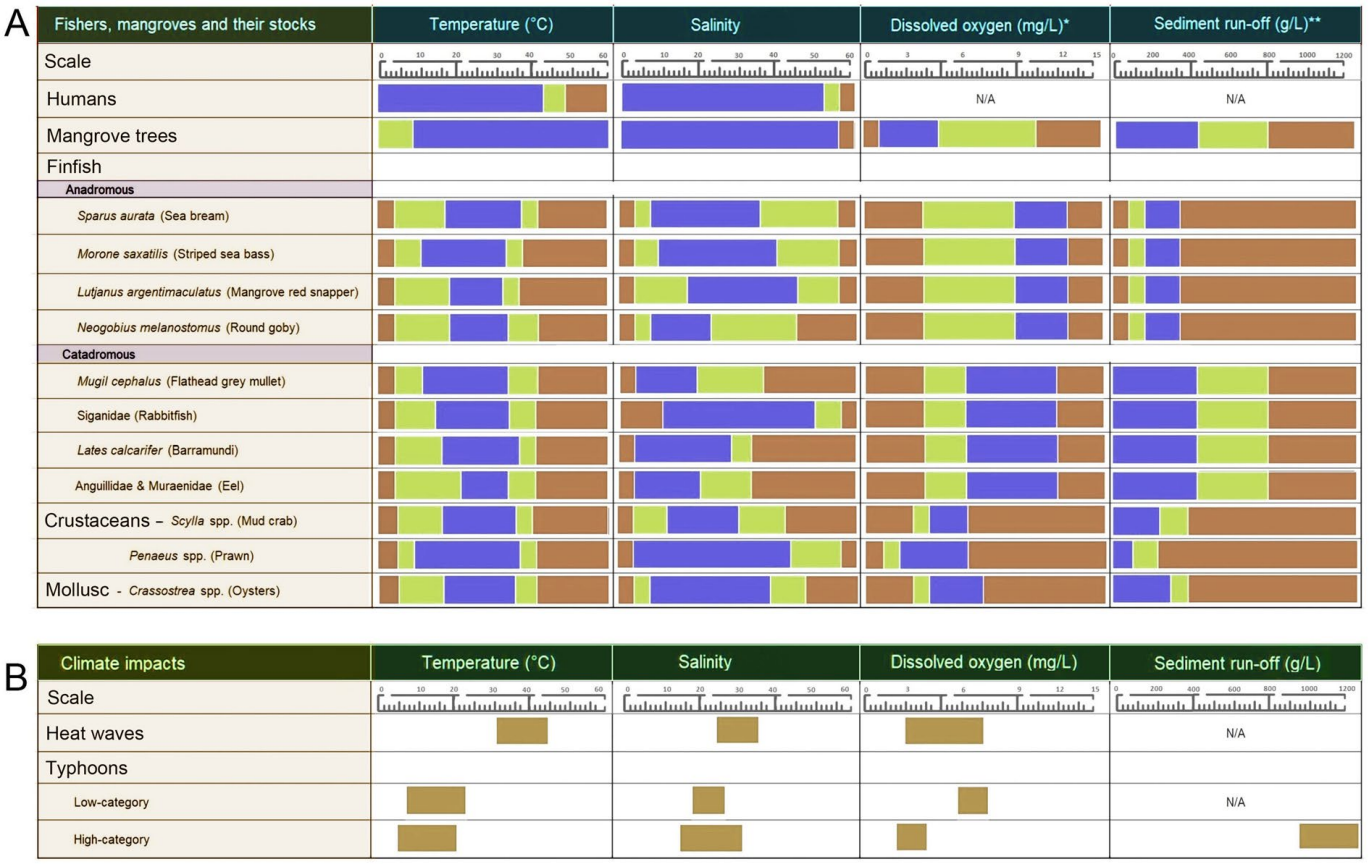
Environmental tolerance levels of mangrove stocks and inhabitants and the predicted shifts of these environmental attributes due to climate impacts. This is a quantitative summary of information provided in the text, and the reader is directed to the references within the text for support. **A** Tolerance ranges for mangroves, stocks, and their fishers. Since there are few data for mangrove stocks, we only include the most commonly caught animals. Thresholds conditions are presented as blue for optimal; orange for sub-lethal; and red for lethal effects. **B** Projected shifts in environmental events due to climate impacts. Bars represent the range of effects each climate impact has on environmental events. N/A indicates that the climate impact has no noticeable influence on the specific environmental event. *Most of the dissolved oxygen levels are similar for each fish category. Current studies lack specificity, with the majority focusing their assessment on either saltwater or freshwater species. Based on the migration patterns and habitats of fishes (anadromous and catadromous), we have assumed that each category requires the same dissolved oxygen level (US EPA 2015). **Given that mangrove fish are found in both estuaries and freshwater, and that most sediment load studies focus on freshwater species, we have inferred that their limits are similar to those of freshwater species (Supplementary Table S2)

In this section, we indicate initially that some climate impacts (henceforth, *impacts*) will have negligible effects on mangrove ecosystems (see *Non-issues*). Then, we briefly explore the potential long-term (decadal) impacts of *Shifts in ocean current patterns*. However, the focus of this review is on the immediate (within the next 10 years) effects of climate change. By assessing the ecophysiological ranges of the key players (i.e., mangrove forests and stocks) in the mangrove ecosystem (Fig. 4), we recognise three main impacts that will have an immediate and pronounced effect on mangrove ecosystems: *heat waves*, *low-category typhoons*, and *high-category typhoons*—further justification, through examples, of our reasoning is presented in the following subsections. Then, using our overview of the stocks and fishers (*Mangrove forests, their subsistence fisheries, and fishers*; Box 1), for each impact we make predictions of the current, annual: (1) extent to which the stocks will be “adversely affected” (i.e., sub-lethally and lethally combined); (2) lethal effects on stocks; and (3) number of fishing-days lost by fishers. The first of these predictions explores the potential wider effects on fishers. We use the second two predictions in our penultimate section, *An estimate of fishing-days lost due to climate-impacts: a case study*, where lethal effects on stocks are converted to our common currency of “fishing-days lost” (for an explanation of how “days lost” was calculated see Box 1).

### Non-issues

Some aspects of climate change may be ignored when addressing their impacts on mangroves, their stocks, and their fishers. Firstly, mangrove forests provide a barrier to physical impacts, protecting the fishers as well as stocks such as fish and prawns that live amongst the inner margins and roots of mangroves, crabs that live in burrows, and oysters that are attached to the roots (Asari et al. 2021). Therefore, only extreme events—ones that will be exacerbated by climate change—require addressing. Mangrove forests grow and accrete sediments at rates that are sufficient to obviate the effects of sea level rise, estimated to be a maximum of 10 mm per year (Krauss et al. 2013; McIvor et al. 2013). Likewise, high organic loads and biogeochemical cycling within the estuary increases water alkalinity, removing threats of ocean acidification (Sippo et al. 2016). In contrast, there are several climate impacts that will affect mangroves and their inhabitants; these are examined below.

### Shifts in ocean current patterns

Here, we briefly examine wind-driven surface currents that span thousands of kilometres, rather than small-scale changes, such as typhoon-driven surges (these are dealt with below, under the section *High-category typhoons*). Such large-scale currents will be subject to long-term (decadal) changes due to climate change (Harley et al. 2006; Wu et al. 2017) and will alter coastal currents, impacting mangroves ecosystems. For example, recent models indicate that rising temperatures could alter the direction and either weaken or strengthen currents (Peng et al. 2022; Vecchi and Soden 2007). However, as our focus is on changes in the next 10 years, these currents are not of direct concern to this review. Here, we simply recognise their importance and need for further study.

Changes in surface currents may have multiple effects. They may cause shifts in phytoplankton biomass and distributions; influence species life cycles (Falkowski 2012; Hays 2017); displace taxa with oceanic stages (i.e., larval, and juvenile fish, crabs, and prawns); and impede homing behaviours of fish (see *Mangrove forests, their subsistence fisheries, and fishers*; van Gennip et al. 2017; Wilson et al. 2016). Unfortunately, there are not any studies on the direct effect of these currents on mangrove stocks. To illustrate the potential effects on coastal ecosystems, we examine some impacts of three example currents. The weakened Kuroshio and North equatorial currents (i.e., in the North Pacific, Atlantic and Indian Oceans) reduced the likelihood of juvenile Japanese eels reaching their estuarine and freshwater nurseries by 40% (Chang et al. 2018, 2019). Furthermore, increased currents, such as the East Australian current, are disrupting marine communities by introducing new populations to remote areas (Phillips et al. 2022; Seebens et al. 2020). For example, the dispersion of green crabs in temperate regions has been associated with these intensified ocean currents, although explicit quantification is not available (Young and Elliott 2019). The arrival of invasive species through such current changes may then have detrimental effects on native populations (Katsanevakis et al. 2014).

Again, while these current shifts clearly merit attention, we have excluded them from our analysis on fishing-days lost (see *An estimate of fishing-days lost due to climate-impacts: a case study*) due to their long-term impacts and a lack of directly relevant data. However, we do return to shifts in currents in our final section (*Outlook*), where we suggest how they might be explored.

### Heat waves

There is virtually no information on the effect of heat waves on mangrove ecosystems, so this section relies on relevant data from comparable systems (Wetz and Yoskowitz 2013). Climate change driven atmospheric heat waves are predicted to increase in frequency, from two to six per year and in intensity reaching > 40 °C for more than five days (Perkins-Kirkpatrick and Gibson 2017). These atmospheric heat waves tend to coincide with estuarine heat waves, which occur two to ten times per year (Tassone et al. 2022); we have assumed that similar frequencies and intensities will apply to mangrove estuaries, suggesting five heat waves per year in *An estimate of fishing-days lost due to climate-impacts: a case study* (below).

The frequency and intensity of heat waves will increase due to climate change, historically evidenced by a 54% increase in the annual global heat wave count from 1925 to 2016 (Oliver et al. 2018). Increase in heat waves over the next decade will, however, be site specific (Perkins-Kirkpatrick and Gibson 2017). As an example, in our analysis (Box 1), using data from the Climate-Analytics Climate Impact Explorer (https://climate-impact-explorer.climateanalytics.org/impacts), we predicted a ~ 30% increase in the number of days affected by heat waves in the Philippines over the next decade (Supplementary Table S9). Such increases threaten coastal ecosystems, e.g., kelp (Smale 2019), seagrass beds (Serrano et al. 2021) and coral reefs (Hoegh-Guldberg et al. 2017). Already, over the last decade, 60% of global marine ecosystems have been degraded due to heat waves, with coral reef and seagrass ecosystems declining by 14% and 30%, respectively (Smale et al. 2019b; United Nations Environment Programme 2020, 2021). Although mangrove ecosystems are more heat-tolerant than coral reefs and sea grass beds they too will be affected (Li et al. 2022).

Mangrove stocks will respond differently to heat waves because of their biological diversity (see *Mangrove forests, their subsistence fisheries, and fishers*). However, all taxa face challenges from heat waves through three key environmental factors: extreme temperatures, increased salinity, and reduced dissolved oxygen (Fig. 4A, B; Tassone et al. 2022; Vinagre et al. 2018). Heat waves will adversely affect (i.e., sub-lethally and lethally) ~ 10%–70% of fish and ~ 5%–90% of prawns, despite their eurythermal and euryhaline natures (Fig. 4A; Box 1, Supplementary Table S5). Elevated temperature and salinity will also adversely affect crabs and oysters by ~ 10%–90% and 35%–80%, respectively (Fig. 4A; Box 1, Supplementary Table S5). For example, the 2013 Australian marine heat wave resulted in a 30%–40% loss of crustacean and mollusc populations (Chandrapavan et al. 2019; Roberts et al. 2019). Furthermore, although we lack quantification for mangroves, heat waves may stimulate growth of harmful phytoplankton, weakening fish and oyster immune systems, leaving them susceptible to microbial infection (Roberts et al. 2019). Much of the information above is from laboratory studies or discrete catastrophic events in the field that, although instructive, may not represent typical in situ losses of stocks. For our evaluation of the direct effects of heat waves on stocks, we used the available literature to conservatively estimate lethal losses (i.e., mortality) due to heat waves as follows: 0% for fish, 3% for crabs, 4% for prawns, and 5% for molluscs (for our reasoning behind these values see Box 1, Supplementary Table S6).

Heat waves will also affect the fishers. For example, elevated temperatures lead to shallow estuarine waters drying up (Vinagre et al. 2018); this will indirectly affect fishers by increasing pollutant concentrations and stagnant waters, reducing potable water (Kubicz et al. 2021) and increasing vector-borne illnesses, such as West Nile and dengue fever (Damtew et al. 2023; Paz 2015, *Subsistence fishers, their homes, health, and technologies*). There are also direct effects. Physically demanding, unshaded fishing and poorly constructed houses exposes fishers to dehydration and heat stroke (Kovats and Hajat 2008; Box 1, Supplementary Table S7), incapacitating ~ 20%–40% of fishers for 2–4 days (ILO 2019; Box 1, Supplementary Table S7). Furthermore, during heat waves, which last ~ 5–10 days, fishing ceases (ILO 2019; Perkins-Kirkpatrick and Gibson 2017). We have assessed the annual loss of fishing-days due to cessation of fishing and sickness to be 10 and 1.2 days, respectively (Box 1, Supplementary Tables S4, S7).

### Low-category typhoons

Low-category typhoons (category 1 and 2) are characterized by wind speeds of ~ 119–177 km/h and days where precipitation ranges from 100 to 500 mm (NHC-NOAA 2023; PAGASA 2021). These typhoons typically last 1 to 2 days and occur with a frequency of two to five times per year (Box 1, Supplementary Table S4). These typhoons lower salinity (~ 1–2), temperature (~ 1–2 °C), and dissolved oxygen (~ 1–2 mg/L) (Cui et al. 2023; Liu et al. 2020; Miao et al. 2023). Historical data indicate a 13% decline in the annual frequency of low-category typhoons between 1900 and 2000 (Chand et al. 2022). Extending this declining trend—which deviates from most other impacts of climate change—we have predicted a ~ 46% decrease in the number of days per year affected by low-category typhoons in the Philippines over the next decade (Box 1, Supplementary Table S9); the relevance of this decline is illustrated in our section, *An estimate of fishing-days lost due to climate impacts: a case study*.

Low-category typhoons have moderate effects on stocks, compared to high-category typhoons. Fish, prawns, and crabs tend to be resilient to the associated small changes in salinity and oxygen (Kültz 2015), both adversely affecting stocks by ~ 4–40%, whereas molluscs, are more vulnerable, with ~ 10%–50% being adversely affected (Box 1, Supplementary Table S5; see also, *Mangrove forests, their subsistence fisheries, and fishers*). Temperature change resulting from low-category typhoons are generally too small to have any effect on stocks, as motile taxa such as fish, prawns, and crabs move away, and oysters can withstand the changes (Box 1, Supplementary Tables S5, S6). The above estimates are from laboratory studies focusing on sublethal effects and may not reflect in situ conditions. Hence, we have relied on existing literature on comparable ecosystems to estimate lethal effects, predicting no fatalities for fish, prawns, and crabs, and only a 1% loss of stocks for oysters during these brief events (Box 1, Supplementary Table S6).

Despite their short durations, low-category typhoons do impact fishers by causing illnesses such as diarrhoea, influenza, and dengue fever; they also prevent fishing during the typhoon (see *Subsistence fishers, their homes, health, and technologies*; Sainsbury et al. 2018; Box 1, Supplementary Table S7). We have, therefore, predicted that low-category typhoons currently result in an annual loss of 14 and 6 fishing-days due to fishing cessation and sickness, respectively (Box 1, Supplementary Tables S4, S7).

### High-category typhoons

Limited data exist on the effect of high-category typhoon (categories 3–5) on mangroves, necessitating estimations based on similar ecosystems. High-category typhoons exhibit wind speeds of > 177 km/h and precipitation of > 500 mm per day (NHC-NOAA 2023; PAGASA 2021). They currently last for 2–4 days and occur with a frequency of one to three per year, yielding severe impacts on mangrove ecosystems (Krauss and Osland 2019; Supplementary Table S4). Climate change has quadrupled the number of high-category typhoons since the 1970s, intensified Southeast Asian typhoons by 12%–15% (Mei and Xie 2016), and increased the frequency of related flooding events by two–threefold (WMO 2021). As a site-specific example, based on local data associated with the Philippines, our climate impact model predicts a ~ 17% rise in the days affected by such typhoons over the next decade (Box 1, Supplementary Table S9).

High-category typhoons may harm mangrove stocks in several ways. They alter salinity (3–5) for ~ 5–12 days, stressing animals (Fig. 4A, B; Wada et al. 2014): mobile stocks (fish and prawns) will be adversely affected by ~ 10%–70%, and sedentary and sessile stocks (crabs and oysters) will be affected by 30%–80% (see *Mangrove forests, their subsistence fisheries, and fishers*; Box 1, Supplementary Table S5). High-category typhoons may also induce cold-shocks by lowering water temperatures by 6–12 °C, affecting tropical species (Fig. 4A, B; Doong et al. 2019). For example, a 12 °C drop stressed ~ 20% of coral and freshwater fish (Abram et al. 2017), and similar changes led to a ~ 50% and 90% decline in crustaceans and oysters, respectively (Büttger et al. 2011; Ren et al. 2021). Furthermore, floodwaters laden with terrestrial pollutants (e.g., sewage), could depress oxygen levels (< 2–3 mg/L) for days or weeks, affecting ~ 5%–70% of mobile and ~ 25%–70% of stationary species (Fig. 4A, B; Hutchins et al. 2020; Manitcharoen et al. 2020; Box 1, Supplementary Table S5). High-category typhoons also increase sediment load (> 1 to 40 g/L) through both coastal and riverine flooding (Milliman and Kao 1996; Talbot et al. 2018), burying mangrove roots by ~ 10 cm and harming the trees and their stocks (Fig. 4A, B; Ellison 1999; Supplementary Tables S2, S3). Increased sediments may adversely affect juvenile and adult fish and prawns by ~ 5%–40% and semi-mobile crabs and sessile molluscs by ~ 20%–90% (see *Mangrove forests, their subsistence fisheries, and fishers*; Box 1, Supplementary Table S5). Drawing on existing literature, we estimated lethal affects to be 3% for fish, 5% for crabs, 7% for prawns, and 10% for oysters (Box 1, Supplementary Table S6).

Also, high-category typhoons adversely affect subsistence fishers due to their previously outlined inherent vulnerabilities (see *Subsistence fishers, their homes, health, and technologies*). Over the last decade, these typhoons have caused over 20,000 deaths within coastal communities, destroyed nine million homes, and incurred over US $10 billion in damages (Supplementary Table S1). Typhoon Haiyan exemplifies this, having destroyed ~ 70% of small-scale fishing gear in the Philippines due to wave action and prolonged water exposure (Monteclaro et al. 2018). These disasters also pose acute health risks. Stagnant floodwaters often overflow sanitation systems, contaminating both the environment and water sources of fishers (CDC 2019). Moreover, prolonged coastal submersion—up to 5–7 days—increases physical injuries and water- and vector-borne diseases (see *Subsistence fishers, their homes, health, and technologies*; CDC 2017; Lee et al. 2020; Box 1, Supplementary Table S7). For example, ~ 90% of the population exposed to typhoon Haiyan contracted diarrhoea (Ventura et al. 2015). Mental health issues, such as depression, anxiety, and PTSD are also prevalent among those exposed to high-category typhoons (Maknawa 2019); e.g., of those exposed to the Chinese typhoon Lekima ~ 50% reported PTSD (Zhen et al. 2021). Furthermore, high-category typhoons, lasting 3–4 days, halt fishing (see *Subsistence fishers, their homes, health, and technologies*; Supplementary Table S4). A synthesis of the above data including estimates of the frequency and duration of high-category typhoons suggests that currently 33 and 8.3 fishing-days are annually lost from fishing cessation and sickness, respectively (for assumptions leading to these estimates see Box 1, Supplementary Tables S4, S7).

### An estimate of fishing-days lost due to climate impacts: a case study (for details see Box 1, Supplementary 2 and 3)

Climate change is increasingly disrupting global fisheries, such as mangrove subsistence fisheries, with acute impacts on communities reliant on the stocks (Cheung et al. 2009). To provide advice for management, we have assessed which climate impacts will have the greatest effect on mangrove subsistence fishers, using data from the literature (primary and grey) and our own survey data from the Philippines (Supplementary 3, 4). Our methods and analysis are fully explained in Box 1 and presented as an annotated Excel spreadsheet in Supplementary 2. Here we provide a brief overview of the analysis and its aims.

We focused on the three main climate impacts (henceforth, *impacts*) that will influence mangrove subsistence fishers (i.e., heat waves, low-category, and high-category typhoons), the environmental events (henceforth, *events*) that arise from them (i.e., changes in salinity, temperature, oxygen, flooding, and sediments), the *stocks* that the fishers harvest (i.e., fish, crabs, prawns, and oysters), and the *fishers* themselves. For details on these impacts, events, stocks, and fishers see the above sections *Mangrove forests, their subsistence fisheries, and fishers* and *Climate impacts on the mangrove subsistence stocks and fishers*.

We have used “fishing-days lost” as a common currency to assess the effects of the impacts (Box 1, Fig. 6). To do so, we first determined the current (baseline) number of the fishing-days lost due to the three impacts; this was determined for each environmental event, independently on both stocks and fishers. By examining stocks and fishers separately, we were able to assess which of these two was affected most—knowing this should help direct management to target mitigation. To focus on the imminent effects of climate change, we predicted the increase in fishing-days lost due to climate change over the next decade, based on RCP data (Representative Concentration Pathways; https://climate-impact-explorer.climateanalytics.org/impacts) and assessed three levels of predicted climate change: high-, low-, mid-estimates, as proposed by the RCP.

**Fig. 5:**
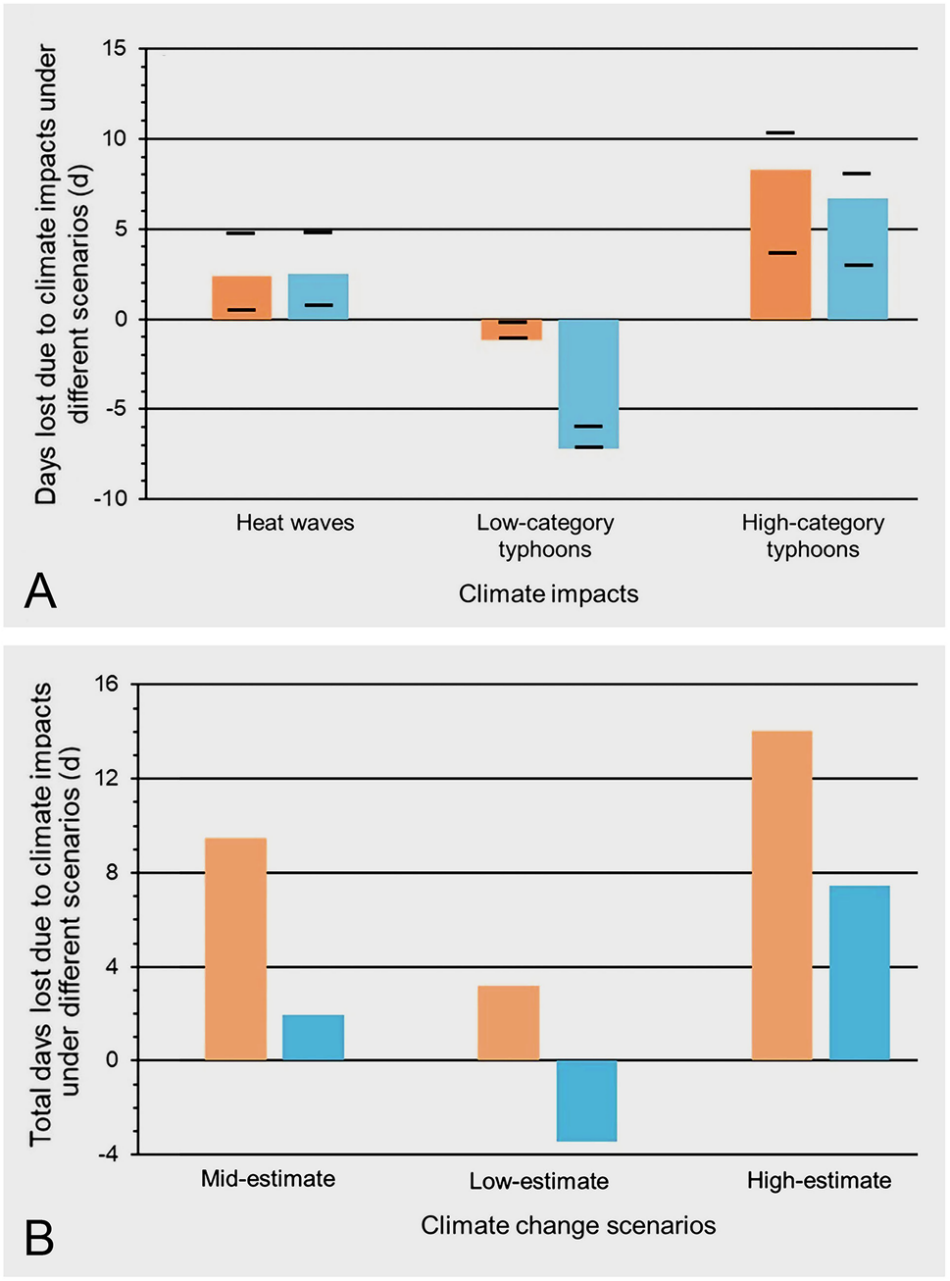
Estimates of fishing-days lost as a result of changes in climate impacts (i.e., heat waves and both categories of typhoons) on stocks (orange bars) and fishers (blue bars). A Effect of changes in the three climate impacts, with coloured bars representing the “expected” RCP-based predictions for the three climate impacts, with the high- and low-predictions indicated by the horizontal black lines. B Combined effects of all three changes due to climate impacts associated with each of the three RCP-based scenarios. The details of these effects, along with the calculations, data sources, and underlying assumptions, are provided in the main text (*An estimate of loss of fishing-days on mangrove subsistence fishers: a case study*) and Box 1

As with all models, ours simplifies complexity by examining what we argue are the main drivers, using the available data, and applying justifiable assumptions (see Box 1; *Climate impacts on the mangrove subsistence stocks and fishers*). In doing so, we offer insights into the potential loss of livelihood to mangrove subsistence fishers and indicate the relative, if not absolute, effects of climate change on the three main impacts on stocks and fishers (i.e., Fig. 5). Critically, our efforts reveal gaps in our knowledge that we discuss below (see *Outlook*).

### Model results

We predicted the number of fishing-days lost over the next decade due to “expected” climate change induced shifts in the three impacts, i.e., heat waves, low-category, and high-category typhoons (coloured bars, Fig. 5A). For heat waves and high-category typhoons there was an increase in the loss of fishing-days, whereas for low-category typhoons there was a decrease in the number of days lost—this decrease is because the number of low-category typhoons is predicted to decrease over the next decade, based on RCP data (Box 1, Supplementary Table S9). The increase in high-category typhoons led to more than twice the number of days lost due to the increase of heat waves. There were distinct differences in the days lost due to effects on stocks and fishers (orange vs blue bars, Fig. 5). The RCP predicted high- and low-estimate of climate change (black horizontal lines, Fig. 5A) provide a range for our predictions of the number of fishing-days lost.

Examining the three RCP climate-change scenarios in more detail (Fig. 5B) revealed that the mid-estimate of climate change resulted in an additional c. 11 lost days (stock and fisher effects combined), the high-estimate nearly doubled this loss (~ 21 days), whereas the low-estimate indicated no days lost (i.e., stock effects were negated by fisher effects). Finally, the effects on stocks and fishers (orange and blue bars, Fig. 5B) indicated a complex interaction between these two affected components, especially when viewed across both impacts and the three levels of possible climate change. For example, fisher-effects accounted for ~ 20% of stock-effects in the mid-estimate, while this increases to ~ 50% for the high-estimate.

### Model evaluation and conclusions

Our analysis is not intended for immediate management decisions but does offer guidance and provides a framework—a model—that can now be elaborated on and then may be useful for future decision making. That said, results from a post-analysis survey of fishers (n = 35) in our study area (Supplementary 3, 4) suggest reasonable agreement with our model parameter estimate of fishing-days per year (264 d) and our baseline estimates of days lost due to heat waves (10 d), low-category typhoons (14 d), and high-category typhoons (33 d). For these, estimates by fishers were, respectively: 239 ± 24 d, 7 ± 2 d, 13 ± 6 d, 30 ± 6 d (± one standard deviation). These findings lend credence to the predictive accuracy of our model and support its continued development.

Our analysis (Fig. 5) indicates that between 11 and 21 fishing-days may be lost due to climate-change induced increases in the three climate impacts (i.e., heat waves, low-category, and high-category typhoons) in the next 10 years; i.e., a loss of 5 to 8% of their current fishing-days. This loss may seem small, but given that most subsistence fishers struggle to survive such a reduction in their ability to collect food may have consequences (Arthur et al. 2021). Furthermore, high-category typhoons had the greatest impact on fishing-days lost, so future analysis should likely focus on them, although heat waves and low-category typhoons must also be considered as they too contribute to the days lost (Fig. 5). Critically, our analysis indicated that there is interaction between all the factors examined: climate impacts, stocks and fishers, and climate change scenarios. These interactions resulted in non-intuitive increases and decreases in the predicted fishing-days lost (Fig. 5).

Given these insights, we recommend further attention to each aspect of our analysis, including synergistic interactions, to enhance our predictions of the impacts of climate change on mangrove subsistence fishers. In the following section, reflecting on both the above analysis and our entire review, we indicate the main gaps in our knowledge and understanding and how they might be filled.

## Outlook

Evidence of the effects of climate change reveals its severe impacts on poor communities worldwide, particularly the subsistence fishery sector (Montejo-Damian et al 2022). Our review identified and quantified the climate impacts that will affect mangrove subsistence stocks and fishers over the next decade. We recognise three main climate impacts—heat waves, low-category typhoons, high-category typhoons—that will directly alter the structure of the mangrove ecosystem, affecting the composition and distribution of stocks and the mangrove subsistence fishers. The consequences of predicted climate-change induced shifts in these impacts, on both stocks and fishers, will lead to a loss in fishing-days (Fig. 5). Critically, our analysis indicates that the effects on stocks and fishers must be examined independently to fully understand how fishing-days are lost. Furthermore, we recognised this loss of days may exacerbate the already vulnerable situation of mangrove subsistence fishers and erode their cultural attachment to the mangrove environment (Maharja et al. 2023). This growing vulnerability underscores the necessity for comprehensive multidisciplinary research to understand and mitigate these effects.

We, therefore, argue that our multidisciplinary approach including models similar to ours, are required to aid management. In this sense, our work serves as a proof of concept that demonstrates more generally the need for multidisciplinary studies to inform climate change mitigation. However, we appreciate that our predictions, based on this first review of how mangrove subsistence fisheries will be affected by climate change, are limited by gaps in our knowledge and understanding—only through review could we recognise these gaps. We conclude this review by briefly identifying five areas that require assessment.

### Improved estimates and parameterisation of harvested stocks

Better estimates of stocks are required to assess the status of fisheries and the impact of climate change. The data in Table 1 and Supplementary Table S6 are our best estimates of mangrove stocks and harvesting methods, but they are based on only a few detailed studies and a handful of global studies, with none directly assessing the effect of environmental conditions from climate change events on mangrove stocks. Our ability to target taxa when conducting ecophysiological assessments (e.g., Fig. 4A) is hampered by a lack of focused studies that detail stocks and their catch. Therefore, we suggest conducting focused assessments of stocks and their capture methods, factoring in the current climate impacts, due to extreme weather, and accounting for climate change projections. This could be achieved using methods such as those employed by CMFRI (2005), Coching et al. (2020), Samoilys and Carlos 2000, and Wolf and Neil (2010).

### Focused ecophysiological studies

Once we understand mangrove stocks better, ecophysiological studies on the main species should be conducted. This is needed for two reasons: (1) to provide better estimates of lethal effects, enhancing the accuracy of our model, and (2) to gain appropriate estimates of sublethal effects, allowing their inclusion in future, more complex iterations of the model. Furthermore, as high-category typhoons are the main impact affecting mangrove subsistence fisheries (Fig. 5), environmental parameters associated with high-category typhoons (Fig. 4b) should be prioritised, potentially by adapting techniques outlined by others: e.g., EPA (2007), Estuary Watch (2020), Klemm et al. (1993), Parsons et al. (1984), US EPA (2002), and Xiaoqing (2003).

### Environmental impacts on mangrove ecosystems

Although the effects of high-category typhoon require attention (Fig. 5), the other main impacts on mangroves—heat waves and low-category typhoons—also need to be better understood (see above, *Climate impacts on the mangrove subsistence stocks and fishers*, Figs. 4B, 5). While extrapolation from similar ecosystems provided some insights associated with the effects of all three impacts, field observations and modelling studies, particularly those recommended by the Estuary Watch (2020), the US EPA (2006), and Vermont Agency of Natural Resources (2022), may now be used to quantify environmental changes in mangrove ecosystems. Furthermore, as indicated above, we know virtually nothing about how shifts in global ocean circulation patterns may affect the dispersal of mangrove stocks (*Shifts in ocean current patterns*). To address this gap, the methods and approaches presented in studies by Fox-Kemper et al. (2019), and Šachl et al. (2019) could offer valuable insights.

### The fishers

As indicated in the section *Climate impacts on the mangrove subsistence stocks and fishers,* there is a dearth of data on the social and health/physiological effects of climate change on mangrove subsistence fishers. Furthermore, as our brief survey suggests (Supplement 3, 4) and others have argued (Hiwasaki et al. 2014; Monteclaro et al. 2018), understanding local fishing technology, the local knowledge that fishers possess, and the perception of fishers related to climate change is paramount to establishing educational methods that will aid in mitigating the effects of climate change; i.e., knowledge of the impacts will be virtually useless unless we can successfully implement adaptation mechanisms, at a grassroots level (Hoang et al. 2022). To this end, surveys and community field studies are required, e.g., using methods of Hiwasaki et al. (2014) and Savaris et al. (2021) to assess social impacts, using methods of Coppola et al. (2011) and Monteclaro et al. (2017) to assess fishing technologies, and using methods of Sansom et al. (2020) to assess health impacts.

Our study also considers the immediate effects of typhoons on the homes of subsistence fishing communities (Healey et al. 2023), highlighting the importance of improving on-land installations for community resilience. Future research should assess the effectiveness of infrastructure improvements in reducing typhoon vulnerability and repair costs, crucial for climate change mitigation strategies. For guiding future studies, methods from Esteban et al. (2010), Fabianova and Estokova (2023), and Wan et al. (2022) can be used to assess housing damage from climate impacts.

### Using “currencies” other than fishing-days lost to assess impacts; i.e., changing our model output

Metrics other than *fishing-days lost* could be examined to assess the impacts of climate change on fishers. Here are three examples that might be pursued: (1) *loss of income*, both direct (market price for stocks) and indirect (gear and consumed stock); (2) *reduction in catch per unit effort* (CPUE), due to reduced stocks; and (3) *loss of fishers* from the community due to unsustainability of the fishing population. Some resources that may offer direction for these further currencies are Appelman (2015) and Macusi et al. (2021) for CPUE analysis and Pulg (2023) for economic impact assessment.

### Box 1: detailed model methods for the case study “An estimate of fishing-days lost due to climate impacts”

**Fig. 6:**
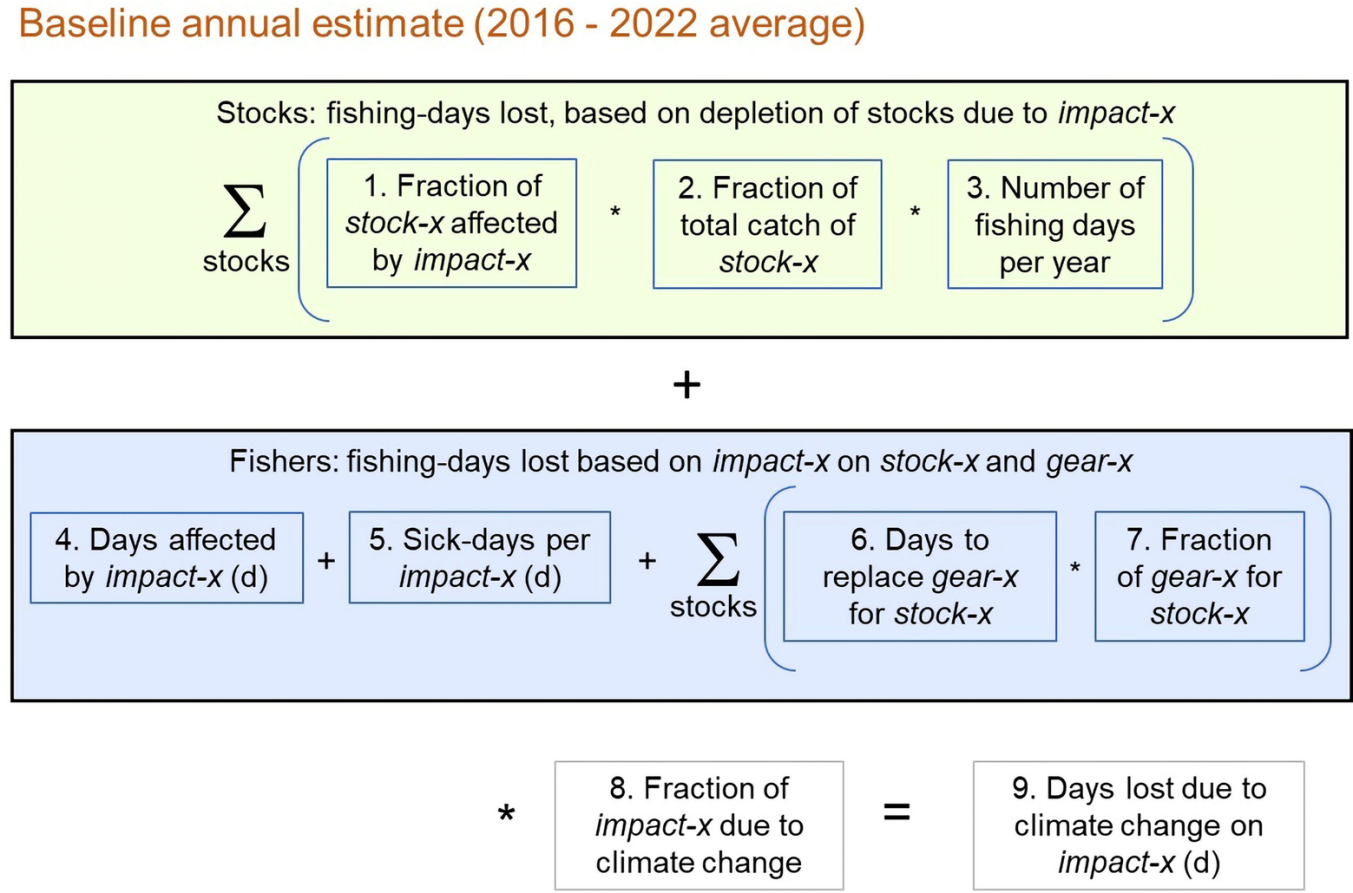
Method used to model the annual fishing-days lost, per fisher, per climate impact. The green box incorporates the effects on stocks, and the blue box incorporates the effects on fishers; details of these effects are presented in the main text and this section. Calculations, data sources, and assumptions associated with panels 1 to 8 (which represent the 8 rectangles in this figure) are detailed in the text of this supplement. *Stock-x* represents one of the four stocks (fish, crabs, prawns, oysters). *Impact-x* represents one of the three main climate impacts (heat waves, low-category typhoons, high-category typhoons). *Gear-x* represents the fishing gear associated with *stock-x*. Fishing occurs 264 days per year (zu Ermgassen et al. 2020)

To assess which climate impact (henceforth, impact) will have the greatest effect on mangrove subsistence fishers we have based our analysis on subsistence fishers from Zambales in the Philippines (E 119° 57.648', N 15° 31.7132') and used data from the literature (primary and grey) and our own survey data from the Philippines (Supplementary 3, 4). Our analysis (Fig. 6) focuses on the three main impacts that will influence mangrove subsistence fisheries, the environmental events that arise from climate change, the stocks that the fishers harvest, and the fishers themselves. We have assumed that the relatively low frequency of the impacts (Supplementary Table S4) means they will not be concurrent in time and can, therefore, be treated as independent events (i.e., their effects are additive).

We have used “fishing-days lost” lost as a common currency to assess which impact has the greatest effect (Fig. 6). To determine the current number of the fishing-days lost due to impacts we have relied on data related to: (1) current estimates of the impacts (see below, *Baseline annual estimates of climate impacts 2016–2022*); (2) the loss of stocks due to each impact (see below, *Stock: fishing-days lost, based on depletion of stocks due to climate impacts*); and 3) the disruption to fishers and the destruction of gear due to each impact (see below, *Fishers: fishing-days lost based on climate impacts on fishers and their gear*). To predict the increase in lost fishing-days due to climate change over the next decade, we multiplied the baseline estimates of days lost by the predicted increase in impacts due to climate change in 2034 (see below, *Estimating the increase in days lost in 2034 for Zambales, Philippines*). Finally, to make the analysis more robust, we assessed three levels of predicted climate change: high-, low-, and mid-estimates as predicted by RCP (see below, *Estimating the increase in days lost in 2034 for Zambales, Philippines*).

Details of the model follow (we have numbered the following sections so that they can be referred to in our annotated spreadsheet that describes the model, Supplementary 2).

#### Baseline annual estimates of climate impacts 2016–2022 (Fig. 6, Supplementary Table S4)

To estimate current (2023) impacts (Supplementary Table S4) we obtained: (1) baseline data (2016 to 2022) on low-category (1–2) and high-category (3–5) typhoons per year (PAGASA ARTC 2018 to 2023a) (PAGASA 2018, 2019, 2020, 2023a) and (2) data on heat waves (Perkins-Kirkpatrick and Gibson 2017; Tassone et al. 2022). Disruptions were based on impact intensity and duration, with high-categories (3–5) typhoons including 5 days of post-typhoon flooding that would prevent fishing (CDC 2017).

#### Stocks: fishing-days lost, based on depletion of stocks due to climate impacts (Fig. 6, panels 1–3)

Data in the literature tend to be presented as fractions of stock-losses due to impacts, and there are laboratory studies that indicate the lethal effect of environmental stressors on stocks; these have been reviewed in the main text (and are summarized in Supplementary Table S5). Although there are few data from field studies, it appears that the realized effects of these impacts in situ (i.e., in marine estuaries such as mangrove forests, coral reefs, seagrass beds) are substantially lower than those observed in the laboratory, often close to an order of magnitude lower (cf. Supplementary Tables S5, S6). Below we summarised our arguments for the loss of stocks due to the impacts that would be observed in nature (these are also presented in Supplementary Table S6). These estimates were then used in the model to predict fishing-days lost (Fig. 6).

For heat waves, fish typically show no mortality as they move to cooler areas (Liao 2007). Crabs exhibit ~ 3% mortality as they can escape heat waves by burrowing (Assan et al. 2020). Prawns are less mobile than fish and exhibit ~ 4% mortality (Motoh 1985). Oysters which are not mobile experience ~ 5% mortality (Masanja et al. 2023; Yang et al. 2016). The pressures exerted by low-category typhoons can also be avoided through escape and, therefore, the effects on stocks parallel those of heat waves. As they are mobile, fish, crabs, and prawns will not be affected (Alberts-Hubatsch et al. 2015; Kunz 2004; Liao 2007; Motoh 1985). However, oysters will experience ~ 1% mortality (Gosling 2004). Finally, high-category typhoons pose greater threats than heat waves and low-category typhoons. Fish experience ~ 3% mortality due to high sediment loads (Bash et al. 2001). Crabs experience ~ 5% mortality from habitat damage and physiological stress (Birtwell 2000). Prawns and oysters experience ~ 7% and ~ 10% mortality, respectively, due to high sediment loads (Poirier et al. 2021).

We have assumed that the recovery time for each stock (i.e., the replenishment of stocks to the previously harvestable state), as a result of the cumulative effects of each impact over the year will be 1 year; e.g., if there are two heat waves each resulting in a loss of stocks, the time for the stock to recover will still be 1 year. In the Philippine context, our reasoning for this is as follows: (1) heat waves and low-category typhoons tend to occur close together, over 1–2 months (PAGASA 2023b, c; Perkins-Kirkpatrick and Gibson 2017; Yin et al. 2022); (2) although high-category typhoons occur over ~ 5 months (PAGASA 2023c), we lack information on the timing of their occurrence; and (3) the time for stocks to recover from the impact varies, ranging around—or just below—1 year (Supplementary Table S6). Consequently, we have simplified the recovery time to 1 year for multiple events, which in some cases will underestimate the recovery time, but in others will overestimate it. Lacking data on the exact timing of events, this estimate of 1 year to recover seems a reasonable assumption.

For the estimates of stock-loss, we have not included any sublethal effects such as reduced growth and reproductive rates, shifts in behaviour, or detrimental ecological effects such as changes in distribution patterns (Dallas and Ross-Gillespie 2015; Komoroske et al. 2016). This is because these effects cannot easily be translated to “fishing-days lost” (Hamel et al. 2023, see below), and there are insufficient data to adequately embed them into the model. We recognise that this assumption may lead to a minor underestimate of the loss of stocks (and hence days lost).

We have then assumed that mean stock-losses can be converted into fractions of fishing-days lost, assuming a linear relationship between catch and stock availability. For example, if heat waves reduce crab stocks by 3% (Supplementary Table S6), we assume that fishers would exert the same effort as if there were no impact. However, 3% of these fishing-days would yield no catch, effectively becoming lost days. This may be an overestimate, as fishers could reduce effort in response to lower stocks, but we lack data to quantify such behaviour.

We further refined our model to account for stock-specific fishing effort variability, by recognizing that the proportion of stocks caught by fishers is not equal (Fig. 6, panel 2). We have, therefore, weighted the fishing-days lost by an estimate of the proportion of different stocks caught by fishers (from Supplementary 3, 4: fish 47%; crabs 24%; prawns 10%; molluscs 19%).

Finally, the number of fishing-days per year of 264 days (Fig. 6, panel 3) represents that of typical artisanal fishers (zu Ermgassen et al. 2020), and this is supported by interviews with local fishers (239 ± 24 d, Supplementary 3, 4); as impacts are determined over 1 year (365 days) the effect of annual impacts on fishing-days was corrected by recognising that 264/365 were affected. Furthermore, to determine the cumulative effects of impacts on lost fishing-days we have assumed that the effect of impacts (i.e., heat waves and typhoons) on stocks are discrete. For instance, if there were two heat waves each leading to a 3% reduction of a given stock, then the first event would reduce the number of fishing-days on that stock to 3% of 264 days = 256 days (assuming the linear relation between catch and effort, see above); then the next event would reduce the 256 days by 3% = 248 days.

#### Fishers: fishing-days lost based on climate impacts on fishers and their gear (Fig. 6, panels 4–7; Supplementary Tables S4, S7, S8)

During a climate impact, fishing was assumed to cease. The number of days lost (Fig. 6, panel 4) was obtained from the product of the frequency and duration of an impact (Supplementary Table S4). In addition, the model accounted for “sick-days” caused by these impacts (Fig. 6, panel 5), applying only the greatest effect (bold values in Supplementary Table S7); i.e., only the illness requiring the longest recovery was considered (Sphere Association 2018), assuming concurrent illnesses did not result in synergistic increases in illness—this may be a conservative estimate*.* Sick-days were calculated as the product of the recovery-days for an illness and its likelihood of occurring, based on literature data (Supplementary Table S7). Note that the days lost for each stock were additive (e.g., if heat waves resulted in 5 days lost in mollusc harvesting and 2 days lost in crustacean harvesting, this would yield a total of 7 fishing-days lost).

Climate impacts will also damage fishing gear (Fig. 6, panels 6, 7), which will be stock specific. Even when gear is not deployed during an impact, it is stored outside and subject to damage from the elements (Anon., pers.com.). We estimated the time to replace gear for fish, crabs, prawns, and oysters, the fraction of gear each fisher has for each stock (Supplementary Table S8; Supplementary 3, 4), and the travel time to buy the materials (Monteclaro et al. 2017). We have assumed that heat waves will damage 5% of the gear, low-category typhoons will damage 10%, and high-category typhoons will damage 50%; these estimates are based on expectations that gear will deteriorate due to heat and be lost through winds and flooding (ICAR 2012; Monteclaro et al. 2017). The product of days to replace the gear and the fraction of gear owned by a fisher (summed over all stocks), provided the number of days lost (Supplementary Table S8).

#### Estimating the increase in days lost in 2034 for Zambales, Philippines (Fig. 6, panel 8; Supplementary Table S9)

The predicted change between 2023 (our baseline, see above) and 2034 was determined for each climate impact (heat waves, low-category typhoons, high-category typhoons). To do so, we used data from the Climate-Analytics Climate Impact Explorer (2023) (https://climate-impact-explorer.climateanalytics.org/impacts), specifically targeting projections for Zambales, Philippines. Data on the three climate impacts were not available. Consequently, we followed recommended practices: heat waves were considered to be days > 38 °C; low-category typhoons (1–2) were considered to be days with precipitation between 100 and 250 mm; high-category typhoons (3–5) were considered to be days with precipitation > 250 mm (Mazdiyasni et al. 2019; PAGASA 2021; Perkins-Kirkpatrick and Gibson 2017; Tassone et al. 2022).

The fractional change in an impact (*I*) was calculated as: (*I*_2034_–*I*_2023_)/*I*_2023_. For a more robust assessment, we extended the analysis to include three scenarios provided by the RCP (Representative Concentration Pathways); for details of these see van Vuuren et al. (2011). The three scenarios were: RCP 2.6, a “low-estimate;” RCP 8.5, a “high-estimate;” and RCP 4.5, a “mid-estimate” (Supplementary Table S9). This approach follows that of others (Pope et al. 2021; Wang et al. 2019).

The number of lost days due to increases in impacts from 2024 to 2034 was calculated as the product of the baseline days lost due to each impact (Fig. 6, panels 1–7) and the fractional change of that impact changing between 2024 to 2034 (Supplementary Table S9; Fig. 6, panel 8). We also calculated the total days lost for each impact by summing the days lost for both stocks and fishers under each climate impact scenario (Supplementary 3).

We then compared the number of days lost due to each impact in the main text (Fig. 5). Our raw data and the associated calculations leading to Fig. 5 are available in the tables associated with this supplement and as an annotated Excel file (Supplementary 2).

## Supplementary Information

Below is the link to the electronic supplementary material.

Supplementary file1 (PDF 317 kb)

## Data Availability

The data sets generated during and/or analysed during the current study are available from the corresponding author (RPA) upon reasonable request.
