# Supplementary material for: Impacts of climate change on mangrove subsistence fisheries: a global review
Source: Mar Life Sci Technol. 2024 Jun 5;6(4):610–30. doi: 10.1007/s42995-024-00231-3 (PMC11602924; doi:10.1007/s42995-024-00231-3)
Supplement: Supplementary file 1 — Supplementary file1 (PDF 317 kb) [file 42995_2024_231_MOESM1_ESM.pdf]

Supplemental material for: Impacts of climate change on mangrove subsistence fisheries: A global review

*Roann P. Alberto[a], Judith A. Teano[b], Annie Melinda Paz-Alberto[c], Mark Anthony B. Tangonan[d], Hazel Jade E. Villamar[e], Sarah Clement[f], *David J. S. Montagnes[g], Andrew P. Morse[h]

*Corresponding authors at: Entrepreneurship, College of Business Administration and Accountancy, Central Luzon State University, Science City of Munoz, Nueva Ecija, 3120, Philippines

Email address: R.P. Alberto@liverpool.ac.uk (Roann Alberto)

*Department of Evolution, Ecology and Behaviour, University of Liverpool, BioSciences Building, Crown Street, Liverpool, L69 7ZB.

E-mail address: dmontag@liv.ac.uk (D.J.S.Montagnes)

[*a]*Entrepreneurship, College of Business Administration and Accountancy, Central Luzon State University, Science City of Munoz, Nueva Ecija, 3120, Philippines*

[b]*Environmental Economics, College of Business Administration and Accountancy, Central Luzon State University, Science City of Munoz, Nueva Ecija, 3120, Philippines*

[c]*Retired University Professor, Ecology and Environmental Management, Institute for Climate Change and Environmental Management, Central Luzon State University, Science City of Munoz, Nueva Ecija, 3120, Philippines*

[d]*Accountancy and Finance, College of Business Administration and Accountancy, Central Luzon State University, Science City of Munoz, Nueva Ecija, 3120, Philippines*

[e]*Accountancy and Finance, College of Business Administration and Accountancy, Central Luzon State University, Science City of Munoz, Nueva Ecija, 3120, Philippines*

[f]*Environmental Policy, Australian National University, Canberra ACT 2601, Australia*

[*g]*Department of Evolution, Ecology & Behaviour, University of Liverpool, BioSciences Building, Crown Street, Liverpool, L69 7ZB, UK*

[h]*School of Environmental Sciences, Roxby Building, University of Liverpool, L69 7ZT, UK*

Supplemental Materials Section

NOTE: Supplements 2 and 4 are annotated Excel files and are presented separately from this document: An estimate of the fishing-days lost due to climate impacts.xlsx and Fishing practices survey on mangrove subsistence fishers in Zambales, Philippines.xlsx.

Supplement 1: this supplement provides support for arguments made in the main text; i.e., the tables below are referred to in the main text.

Table S1 Typhoons and their effects on coastal communities. These data are not exhaustive and do not include all historical typhoons, cyclones, and hurricanes; sources are presented as numbered footnote associated with the typhoon name. Inclusion is limited to typhoon categories 4 and 5, that have caused major devastation to coastal communities between 2013-2023. The Saffir-Simpson scale was used to determine wind speed (NHC NOAA 2023 - https://www.nhc.noaa.gov/aboutsshws.php).
[1]ADRC (2019), [2]Aguilar-Roman et al. (2020), [3]Aquino et al. (2018), [4]Business Standard (2016), [5]Cahigas et al. (2023), [6]Chauhan et al. (2020), [7]DFAT (2021), [8]Ezer (2020), [9]Hallwright and Handmer (2019), [10]Keriwala and Patel (2022), [11]Kishore et al. (2018), [12]Kumar et al. (2021), [13]Li and Huang (2018), [14]Mangubhai et al. (2021), [15]Marlier et al. (2022), [16]Monteclaro et al. (2018), [17]Nair (2021), [18]NHC-NOAA (2014), [19]NHC-NOAA (2018a), [20]NHC-NOAA (2018b), [21]NHC-NOAA (2020), [22]NPR (2018), [23]OCHA (2020), [24]Parida et al. (2018), [25]Reliefweb (2015), [26]Reliefweb (2017), [27]Reliefweb (2019), [28]Reliefweb (2023), [29]Santos et al. (2016), [30]Santos (2021), [31]Senkbeil et al. (2020), [32]UNESCAP (2021), [33]Wingard et al. (2019), [34]World Bank (2014), [35]World Bank (2015), [36]World Vision (2020)

Name	Year	Category	Wind speed ($m\ s^{-1}$)	Location	Storm surge height (m)	Storm surge Damage Scale	Homes Destroyed (x1000)	Deaths
North Atlantic								
Irma[15,33,36]	2017	Category 5	79	United States/ Caribbean	0.9-1.5	Minimal	6,000	129
Maria[11,15,22]	2017	Category 5	78	Puerto Rico	1.8-2.7	Extensive	10	4,000
Michael[19,31]	2018	Category 5	72	Florida USA	4.6	Extreme	180	16
Dorian[8,28]	2019	Category 5	82	Bahamas	1.5	Minimal	76	74
Iota[21]	2020	Category 4	69	Central America	4.6-6.1	Catastrophic	63	84
Eastern Pacific Ocean								
Odile[18]	2014	Category 4	1	Mexico	1.8-3	Moderate	10	5
Patricia[2]	2015	Category 5	98	Mexico, Central America	3	Extensive	9	13
Willa[20]	2018	Category 5	72	Mexico	2.7	Extensive	7	9
Western Pacific Ocean								
Haiyan[16,29,30]	2013	Violent typhoon	64	Philippines	7.6	Catastrophic	1,100	7,000
Vongfong[23]	2014	Violent typhoon	60	Philippines	1.8-4.0	Extensive	180	2
Soudelor[13,25]	2015	Violent typhoon	60	Eastern China, Taiwan	14.63	Catastrophic	69	33
Meranti[4]	2016	Violent typhoon	61	China, Taiwan	2.1-3.0	Extensive	331	28
Haima[19,26,32]	2016	Violent typhoon	60	Philippines	4.6	Extreme	200	14
Rai[5]	2021	Violent typhoon	61	Philippines	3	Extensive	400	410
North Indian Ocean								
Phailin[24,34]	2013	Extremely severe cyclonic storm	60	India	6.1	Catastrophic	500	10,000
Hudhud[1]	2014	Extremely severe cyclonic storm	51	Nepal/India	7.9	Catastrophic	920	45
Fani[6,27]	2019	Extremely severe cyclonic storm	60	India, Bangladesh	1.2-1.5	Minimal	189	89
Amphan[12]	2020	Super cyclonic storm	67	India, Bangladesh	10.36	Catastrophic	1,000	84
Tauktae[10,17,32]	2021	Extremely severe cyclonic storm	51	India, Maldives, Pakistan, Sri Lanka	3.4	Extensive	56	67
South Pacific Ocean								
Pam[9,35]	2014	Category 5	69	Vanuatu	13.41	Catastrophic	188	11
Winston[3]	2015	Category 5	78	Fiji	3	Moderate	40	44
Harold[7,14]	2019	Category 5	64	Fiji	3	Moderate	180	31

Table S2 The effect of suspended sediment levels on fish, crustaceans, and molluscs. Estimates are based on freshwater species (Birtwell 2000), and the criteria are neither species-specific nor life stage specific.

Suspended sediment load (g L^{-1})	Risk to fish and their habitats
0	No risk
<25	Very low risk - No evidence of harmful effects on stocks
25-100	Low risk - It should be possible to maintain moderately healthy stocks, but the yield would be somewhat diminished
100-200	Moderate risk - These waters are unlikely to support healthy stocks
200-400	High risk - at best, only poor fisheries are likely to be found
>400	Unacceptable risk - mortality

Table S3 Typhoon-related flooding events that have caused mangrove root burial and mortality. Sources are presented as numbered footnote associated with the typhoon name.

Typhoon/Hurricane	Year	Location	Sediment Deposition affecting the mangrove roots (cm)
Donna	1962	USA	12-13[3]
Ofa	1990	American Samoa	13[3]
Mitch	1998	Central America	100[2]
Wilma	2005	USA	8-10[4]
Haiyan	2013	Philippines	1000[1]
Irma	2017	USA	12[4,5]

[1]Brill et al. (2016), [2]Cahoon et al. (2003), [3]Ellison (1999), [4]Feher et al. (2019), [5]Wingard et al. (2019)

Table S4 Frequency (per year) and duration (days) of heat waves and typhoons, and an estimate of the fishing-days directly lost per year by these (i.e., the product of frequency and duration). We assumed that high-category typhoons produced flooding that led to five further days lost (CDC 2017). Total days (d) are obtained by adding the fishing days directly lost (d) to flood-affected days (d). See *Baseline annual estimates of climate impacts 2016-2020* for details.

	**Frequency (per year)**	**Duration (d)**	**Fishing days directly lost (d)**	**Flood-affected days (d)**	**Total days (d)**
Heat waves	2	5	10		**10**
Low-category typhoon					
Category 1	5	2	10		10
Category 2	2	2	4		4
					**14**
High-category typhoons					
Category 3	1	3	3	5	8
Category 4	3	4	12	5	17
Category 5	1	3	3	5	8
					**33**

Table S5 Possible loss of stocks by environmental events caused by discrete climate impacts. Losses are in decimal fractions of the stock lost, and ranges are from data across the literature (denoted by superscripts). For details see, *Stock: fishing-days lost, based on depletion of stocks due to climate impacts*.

Impact	Effects	Stock	Loss range
Heat waves			
	Salinity	Fish	0.10-0.50[3,36,37]
		Crabs	0.20-0.40[14,18,28]
		Prawns	0.20-0.65[2,27,38]
		Oysters	0.50-0.80[21,32,35]
	Temperature	Fish	0.30-0.70[11,24,26,34]
		Crabs	0.10-0.90[9,10,26,29,34]
		Prawns	0.10-0.90[17,34]
		Oysters	0.35-0.60[9,10.21,32,35]
	Oxygen	Fish	0.10-0.72[23.25,31]
		Crabs	0.30-0.50[14,16]
		Prawns	0.05-0.50[1,27,38]
		Oysters	0.25-0.70[4,13]
Low-category typhoons			
	Salinity	Fish	0.04-0.40[3,36]
		Crabs	0.10-0.30[14,18,28]
		Prawns	0.10-0.40[2,27,38]
		Oysters	0.10-0.50[15,32,35]
	Oxygen	Fish	0.10-0.30[23,25,31]
		Crabs	0.10-0.30[14,16]
		Prawns	0.05-0.30[1,27,38]
		Oysters	0.10-0.20[4,13,20]
High-category typhoons			
	Salinity	Fish	0.10-0.40[3,36]
		Crabs	0.30-0.70[14,18,28]
		Prawns	0.20-0.70[2,27,38]
		Oysters	0.50-0.80[15,21,32,35]
	Sediment	Fish	0.05-0.40[6,7,22]
		Crabs	0.20-0.80[7,22,33]
		Prawns	0.05-0.25[8,19]
		Oysters	0.50-0.90[7,12,22,30]
	Oxygen	Fish	0.10-0.72[23,25,31]
		Crabs	0.30-0.50[14,16]
		Prawns	0.05-0.50[1,27,38]
		Oysters	0.25-0.70[4,13,20]

[1]Allan and Maguire (1991), [2]Allan and Maguire (1992), [3]Bachman and Rand (2008), [4]Baker and Mann (1992), [6]Bash et al. (2001), [7]Birtwell (2000), [8]Bommireddy et al. (2021), [9]Caputi et al. (2016), [10]Caputi et al. (2019), [11]Cheung and Frolicher (2020), [12]Colden and Lipcius (2015), [13]Coxe (2022), [14]Davenport and Wong (1987), [15]Des et al. (2021), [16]Faturrohman et al. (2017), [17]Hewitt and Duncan (2002), [18]Ji et al. (2022), [19]Kathyayani et al. (2019), [20]Long et al. (2008), [21]Lowe et al. (2017), [22]Lunt and Smee (2014), [23]McNatt and Rice (2004), [24]Mora and Ospína (2001), [25]Plante et al. (2005), [26]Roberts et al. (2019), [27]Rosas et al. (1998), [28]Ruscoe et al. (2004), [29]Sanda et al. (2022), [30]Sanders et al. (2015), [31]Seager et al. (2000), [32]Sehlinger (2018), [33]Shives and Dunbar (2010), [34]Vinagre et al. (2015), [35]Wang and Li (2018), [36]Whitfield et al. (2006), [37]Young et al. (2022), [38]Zhang et al. (2006).

Table S6 Estimates of in situ losses caused by discrete climate impacts and the recovery times of stocks after these (i.e., values applied in the model). Values are: 1) the fraction of a stock lost per event (see *Stocks: fishing-days lost, based on depletion of stocks due to climate impacts*) and 2) the fraction of a year for a stock to reach harvestable sizes, after stock losses (see footnotes for sources).

Impact	Stocks	Fraction of stock lost per event	Fraction of a year to reach harvestable size
Heat waves			
	Fish	0	0.77[5,7,8]
	Crabs	0.03	0.61[6,12]
	Prawn	0.04	0.53[3,4,9]
	Oysters	0.05	1.19[1,2,10,11]
Low-category typhoons			
	Fish	0	0.76[5,7,8]
	Crabs	0	0.60[12]
	Prawn	0	0.52[3,4,9]
	Oysters	0.01	1.18[1,2]
High-category typhoons			
	Fish	0.03	0.86[5,7,8]
	Crabs	0.05	0.69[12]
	Prawn	0.07	0.61[3,4,9]
	Oysters	0.1	1.28[1,2,10,11]

[1]Barman et al. (2022), [2]Bordignon et al. (2020), [3]Boyd et al. (2002), [4]Briggs et al. (2004), [5]Britten et al. (2017), [6]Chandrapavan et al. (2019), [7]Dinh et al. (2022), [8]FAO (2009a), [9]FAO (2009b), [10]Hansten (2017), [11]Hong et al. (2022), [12]Jumawan et al. (2021)

Table S7 Sick-days arising from the most common illnesses due to discrete climate impacts. The illness with the longest duration is in bold (i.e., the value used in the model, see *Fishers: fishing-days lost based on climate impacts on fishers and their gear*). Sick-days were calculated as the product of the recovery days of an illness and its likelihood of occurring, based on literature data (see footnotes for sources).

Impact	Disease/Illness	Likelihood	Recovery-days (d)	Sick days (d)
Heat waves				
	Dehydration	0.50	2	1.0[10,18]
	Heat stroke	0.18	7	**1.2**[3,11,19]
Low-category typhoons				
	Diarrhoea	0.96	3	2.9[14,22]
	Influenza	0.85	7	**6.0**[13]
	Dengue fever	0.67	7	4.7[7,9,16]
High-category typhoons				
	Influenza	0.85	7	6.0[13]
Water-borne	Cholera	0.97	6	5.8[2,24]
	Diarrhoea	0.96	3	2.9[14,22]
	Leptospirosis	0.59	14	**8.3**[5,21]
	Hepatitis A	0.61	7	4.3[6,12,23]
	Typhoid fever	0.24	7	1.7[1,8,17]
Vector-borne				
	Dengue fever	0.67	7	4.7[7,9,16]
	Malaria	0.074	7	0.5[8,20]
Physical Injury				
	Cuts and contusions	0.10	4	0.4[4,15]
	Lacerations	0.60	21	0.1[4]
	Fractures	0.01	56	0.56[4]

[1]Akhir et al. (2018), [2]Ali et al. (2015), [3]Arsad (2019), [4]Bartholdson and von Schreeb (2018), [5]CDC (2017), [6]CDC (2020), [7]CDC (2021), [8]CDC (2022), [9]Chan and Johansson (2012), [10]Cleveland clinic (2023), [11]Davis (2022), [12]Franco et al. (2012), [13]Go Cheng et al. (2020), [14]Health Australia (2023), [15]Knight (2008), [16]Li et al. (2021), [17]Liu et al. (2018), [18]Manning (2007), [19]Mastrangelo et al. (2007), [20]Nsereko et al. (2020), [21]Rajapakse (2022), [22]Ventura et al. (2015), [23]WHO (2010), [24]WHO (2022).

Table S8 Estimated days to replace damaged fishing gear after it had been destroyed by a discrete climate impact. For details see *Fishers: fishing-days lost based on impacts on fishers and their gear*.

Stock	Gear	Fraction of gear per stock	Days to replace gear (d)
Fish			
	Gillnets	0.5	4
Crabs			
	Crab pots	0.22	1
Prawns	Scoop net	0.07	2
Oysters	Rakes/scythe	0.21	0.5

Table S9 Three scenarios of predicted change of climate impacts on mangrove ecosystems in Zambales, Philippines for 2030 (see *Estimating the increase in days lost in 2030 for Zambales, Philippines*). RCP values outline future climate scenarios based on varying levels of greenhouse gases, affecting how much heat is trapped by atmosphere (van Vuuren et al. 2011). Values are expressed as fractions that denote the increase due to climate change, above our baseline values. Note, that negative values denote a decrease in the current impacts.

**Impact scenario**	**Projected fractional change between 2020 and 2030**
Heat waves	
Mid-estimate (RCP 4.5)	0.30
High-estimate (RCP 8.5)	0.60
Low-estimate (RCP 2.6)	0.12
Low-category typhoons	
Mid-estimate (RCP 4.5)	-0.46
High-estimate (RCP 8.5)	-0.37
Low-estimate (RCP 2.6)	-0.46
High-category typhoons	
Mid-estimate (RCP 4.5)	0.17
High-estimate (RCP 8.5)	0.21
Low-estimate (RCP 2.6)	0.07

Supplement 2: an annotated Excel file of the model describe in Supplement 2 (An estimate of the fishing-days lost due to climate impacts).

Supplement 3: fishing practices survey on mangrove subsistence fishers in Zambales, Philippines.

To augment published data, we conducted a survey to analyse the practices of mangrove subsistence fishers (n = 35) in Zambales, Philippines (E 119° 57.648', N 15° 31.7132'), which is a typical mangrove ecosystem that supports a wide range of fishing activities (Empeno and Gregorio 2015). The survey assessed: 1) the average mass and daily quantity of their catch; 2) the cost and amount of fishing gear; 3) the market price of commonly caught stocks; 4) the number of fishing days per month; 5) the duration of daily fishing activity; 6) the duration of daily active fishing hours; and 7) the days lost per year due to heat waves, low-category, and high-category typhoons. Data were collected between July and September 2023 by telephone interviews lasting 15 to 30 minutes (see Supplement 4, *Fishing practices survey on mangrove subsistence fishers in Zambales, Philippines*).

From the average mass *(M)* of stocks caught, we calculated the proportion of each individual stock – fish, crabs, prawns, and oysters – that each fisher catches, relative to the total mass of the catch, using the formula: proportion $=M_{stock}/M_{total}$, where M_{stock} and M_{total} represent the mass of the individual stock and the total mass of all stocks, respectively (Supplement 5, *Survey on the daily catch and gear utilised by mangrove subsistence fishers*). Similarly, from the gear data, we determined the proportion of gear (*G*) lost for each stock based on our survey of subsistence fishers using the same formula: Proportion $=G_{stock}/G_{total}$, where G_{stock} and G_{total} denote the number of gear lost for each stock and the total gear lost across all stocks. Additional insights were gathered on the types of mangrove stocks commonly caught, the preferred fishing techniques, and other useful information such as the effect of impacts on gear damage.

From the data collected, we calculated mean effects and their standard deviations. We also present average, maximum, and minimum responses, these are not included in the analysis (see *Statistical summary of response, Fishing practices survey on mangrove subsistence fishers in Zambales, Philippines*). Lastly, the percentage difference between literature values and fishers' data serves as a validity check against established research, which we refer to in the main text see *Impacts of climate change on mangrove subsistence fisheries: A global review*.

Supplement 4: an annotated excel file of the survey results in Supplement 3 (*Fishing practices survey on mangrove subsistence fishers in Zambales, Philippines*).

References:

ADRC (2019) Disaster information: Cyclone Hudhud. Asian Disaster Reduction Center. https://www.adrc.asia/view_disaster_en.php?NationCode=&Lang=en&Key=1930. Accessed 11 February 2023

Aguilar-Román E, Castillo A, Güiza F (2020) Vulnerability and risk management after hurricane Patricia in a rural community on the Jalisco coast, Mexico. Int J Disaster Risk Reduc 45:1-17 https://doi.org/10.1016/j.ijdrr.2019.101464

Alberts-Hubatsch H, Lee SY, Meynecke JO, Diele K, Nordhaus I, Wolff M (2015) Life-history, movement, and habitat use of *Scylla serrata* (Decapoda, Portunidae): current knowledge and future challenges. Hydrobiologia 763:5–21. https://doi.org/10.1007/s10750-015-2393-z

Akhir MYM, Nor FM, Ibrahim MI, Shafel MN (2018) Association between environmental factors and typhoid fever post massive flood in Northeastern Malaysia. World Appl Sci J. 36: 799-805. https://doi.org/10.5829/idosi.wasj.2018.799.805

Ali M, Nelson AR, Lopez AL, Sack DA (2015) Updated global burden of Cholera in endemic countries. PLoS Negl. Trop Dis 9:1-13. https://doi.org/10.1371/journal.pntd.0003832

Allan GL, Maguire GB (1991) Lethal levels of low dissolved oxygen and effects of short-term oxygen stress on subsequent growth of juvenile *Penaeus monodon*. Aquac 94:27-37. https://doi.org/10.1016/0044-8486(91)90126-R

Allan GL, Maguire GB (1992) Effects of pH and salinity on survival, growth, and osmoregulation in *Penaeus monodon* Fabricius. Aquac 107:33-47. https://doi.org/10.1016/0044-8486(92)90048-P

Aquino DH, Wilkinson SJ, Raftery G, Potangaroa R, Chang-Richards A (2018) Challenges to building housing resilience: the case of Fiji post-cyclone Winston. Procedia Eng 212:475–480. https://doi.org/10.1016/j.proeng.2018.01.061

Arsad AA (2019) Heat related illness. Saudi Med J 40:1195-1201. https://doi.org/10.15537/smj.2019.12.24727

Bachman P, Rand GM (2008) Effects of salinity on native estuarine fish species in South Florida. Ecotoxicol 17: 591-597. https://doi.org/10.1007/s10646-008-0244-7

Baker SM, Mann R (1992) Effects of hypoxia and anoxia on larval settlement, juvenile growth, and juvenile survival of the oyster *Crassostrea virginica*. Biol Bull 182:265-269. https://doi.org/10.2307/1542120

Barman AC, Wong NLWS, Karim MMA (2022) Reproductive cycle of the oyster *Crassostrea* (*Magallana) saidii* (Wong and Sigwart, 2021) from Southeast Asia. Aquac Fish 2022:1-10. https://doi.org/10.1016/j.aaf.2022.05.007

Bartholdson S, von Schreeb J (2018) Natural disasters and injuries: What does a surgeon need to know? Curr Trauma Rep 4:103-108. https://doi.org/10.1007/s40719-018-0125-3

Birtwell IK (2000) The effects of sediment on fish and their habitat. DFO Pacific region. https://waves-vagues.dfo-mpo.gc.ca/library-bibliotheque/255660.pdf. Accessed 12 December 2022

Brill D, May SM, Engel M, Reyes M, Pint A, Opitz S, Dierick M, Gonzalo LA, Esser S, Brückner H (2016) Typhoon Haiyan's sedimentary record in coastal environments of the Philippines and its palaeotempestological implications. Nat Hazards Earth Syst Sci 16:2799–2822. https://doi.org/10.5194/nhess-16-2799-2016

Bommireddy S, Kumar SK, Prasad A, Rao CA (2021) Influence of turbidity on survival of white leg shrimp (*Litopenaeus vannamei*) grow out phase in SPSR Nellore district, Andhra Pradesh, India. J Bio Innov 10:1-9. https://doi.org/10.46344/JBINO.2021.v010i01.01

Bordignon F, Zomeno C, Xiccato G, Birolo M, Pascual A, Trocino A (2020) Effect of emersion time on growth, mortality and quality of Pacific oysters (*Crassostrea gigas,* Thunberg 1973) reared in a suspended system in a lagoon in Northern Italy. Aquac 528:1-9. https://doi.org/10.1016/j.aquaculture.2020.735481

Boyd CE, Hargreaves JA, Clay JW (2002) Shrimp farming and the environment: Codes of practice and conduct of marine shrimp aquaculture. World Bank, NACA, WWF and FAO Consortium Program, USA

Briggs MRP, Funge-Smith S, Phillips M, Subasinghe R (2004) Introductions and movement of *Penaeus vannamei* and *Penaeus stylirostris* in Asia and the Pacific. Food and Agriculture Organization of the United Nations. Bangkok

Britten GL, Dowd M, Kanary L, Worm B (2017) Extended fisheries recovery timeline in a changing environment. Nat Commun 8:1-7. https://doi.org/10.1038/ncomms15325

Business Standard (2016) Typhoon Meranti kills 28 in China. Business Standard. https://www.business-standard.com/article/news-ians/typhoon-meranti-kills-28-in-china-116091700657_1.html. Accessed 13 March 2023

Cahigas MML, Prasetyo YT, Persada SF, Nadlifatin R (2023) Examining Filipinos' intention to revisit Siargao after super typhoon Rai 2021 (Odette): An extension of the theory of planned behavior approach. Int J Disaster Risk Reduc 84:1-19. https://doi.org/10.1016/j.ijdrr.2022.103455

Cahoon DR, Hensel P, Rybczyk J, McKee KL, Proffitt CE, Perez BC (2003) Mass tree mortality leads to mangrove peat collapse at Bay Islands, Honduras after hurricane Mitch. J Ecol 91:1093–1105. https://doi.org/10.1046/j.1365-2745.2003.00841.x

Caputi N, Kangas M, Denham A, Feng M (2016) Management adaptation of invertebrate fisheries to an extreme marine heat wave event at a global warming hot spot. Ecol Evol 6:3583-3593. https://doi.org/10.1002/ece3.2137

Caputi N, Kangas M, Chandrapavn A, Feng M, Hart A, Marin M, de Lestang S (2019) Factors affecting the recovery of invertebrate stocks from the 2011 western Australian extreme marine heat wave. Front Mar Sci 6:1-18. https://doi.org/10.3389/fmars.2019.00484

CDC (2017) Leptospirosis: Signs and symptoms. Centre for Disease Control and Prevention. https://www.cdc.gov/leptospirosis/symptoms/index.html#:~:text=The%20illness%20lasts%20from%20a,recovery%20may%20take%20several%20months. Accessed 30 July 2023

CDC (2020) Viral hepatitis. Centre for Disease Control and Prevention. https://www.cdc.gov/hepatitis/hav/afaq.htm#:~:text=Adults%20are%20more%20likely%20to,as%20long%20as%206%20months. Accessed 30 July 2023

CDC (2021) Dengue: Signs and symptoms. Centre for Disease Control and Prevention. https://www.cdc.gov/dengue/symptoms/index.html#:~:text=Symptoms%20of%20dengue%20typically%20last,recover%20after%20about%20a%20week. Accessed 30 July 2023

CDC (2022) Malaria: Disease. Centre for Disease Control and Prevention. https://www.cdc.gov/malaria/about/disease.html. Accessed 31 July 2023

Chan M, Johansson MA (2012) The incubation periods of Dengue viruses. PLoS One 7:1-7 https://doi.org/10.1371/journal.pone.0050972.

Chandrapavan A, Caputi N, Kangas MI (2019) The decline and recovery of a crab population from an extreme marine heat wave and a changing climate. Front Mar Sci 6:1-12. https://doi.org/10.3389/fmars.2019.00510

Chauhan A, Dash P, Singh R, Kumar R (2020) Impact of tropical cyclone "Fani" on land, ocean, atmospheric and meteorological parameters. Mar Pollut Bull 162:1-15. https://doi.org/10.1016/j.marpolbul.2020.111844

Cheung WWL, Frolicher TL (2020) Marine heat waves exacerbate climate change impacts for fisheries in the northeast Pacific. Sci Rep 10:1-10. https://doi.org/10.1038/s41598-020-63650-z

Cleveland clinic (2023) Dehydration. Cleveland clinic. https://my.clevelandclinic.org/health/treatments/9013-dehydration. Accessed 01 August 2023

Climate Impact Explorer (2023) Climate impacts. https://climate-impact-explorer.climateanalytics.org/impacts/?region=PHL&indicator=tasmaxAdjust&scenario=rcp85&subregion=PH.PN&warmingLevel=1.5&temporalAveraging=annual&spatialWeighting=area&compareYear=2030. Accessed 15 August 2023

Colden A, Lipcius RN (2015) Lethal and sublethal effects of sediment burial on the eastern oyster, *Crassostrea virginica*. Mar Ecol Prog Ser 527:105-117. https://doi.org/10.3354/meps11244

Coxe NC (2022) Effects of hypoxia and high temperature on eastern oysters: investigating differential tolerance in populations and ploidies. Master's Theses. Louisiana State University.

Davenport J, Wong TM (1987) Responses of adult mud crabs (*Scylla serrata* Forskål) to salinity and low oxygen tension. Comp biochem Physiol Mol Part A 86:43-47. https://doi.org/10.1016/0300-9629(87)90274-X

Davis CPD (2022) Heat stroke (Sunstroke). Emedicine health: First aid and injuries centre. https://www.emedicinehealth.com/heat_stroke/article_em.htm. Accessed 01 August 2023

Des M, Fernandez-Novoa D, deCastro M, Gomez-Gesteria JL, Sousa MC, Gomez-Gesteria M (2021) Modeling salinity drop in estuarine areas under extreme precipitation events within a context of climate change: Effect on bivalve mortality in Galician Rías Baixas. Sci Total Environ 790:1-11. https://doi.org/10.1016/j.scitotenv.2021.148147

DFAT (2021) Tropical cyclone Harold. Australian Government Department of Foreign Affairs and Trade. https://www.dfat.gov.au/crisis-hub/tropical-cyclone-harold. Accessed 22 March 2023

Dinh QM, Truong NT, Duc Nguyen TH, Huyen Tran LT, Kieu Nguyen TT, Phan LH (2022) Variations in length-weight relationship, growth, and body condition of the commercial mullet *Ellochelon vaigiensis* in the Vietnamese Mekong Delta. Heliyon 8:1-8. https://doi.org/10.1016/j.heliyon.2022.e11789

Ellison JC (1999) Impacts of sediment burial on mangroves. Mar Pollut Bull 37:420–426. https://doi.org/10.1016/s0025-326x(98)00122-2

Empeno OE Jr., Gregorio OE (2015) State of the mangroves in Masinloc, Zambales. In: Salmo III SG, Favis AMT, Ting MNS, State of mangrove summit: Northwestern Luzon proceedings. Ateneo de Manila University, Quezon City

Ezer T (2020) The long-term and far-reaching impact of hurricane Dorian (2019) on the Gulf Stream and the coast. J Mar Sci 208:1-15. https://doi.org/10.1016/j.jmarsys.2020.103370

FAO (2009a) Cultured aquatic species fact sheets: *Mugil cephalus* (Linnaeus, 1758). https://www.fao.org/fishery/docs/CDrom/aquaculture/I1129m/file/en/en_flatheadgreymullet.htm. Accessed 05 August 2023

FAO (2009b) Cultured aquatic species fact sheets: *Penaeus vannamei* (Boone, 1931). https://www.fao.org/fishery/docs/DOCUMENT/aquaculture/CulturedSpecies/file/en/en_whitelegshrimp.htm. Accessed 05 August 2023

Faturrohman K, Nirmala K, Djokosetiyanto D, Hastuti YP (2017) The concentration of optimum dissolved oxygen levels for growth of mangrove crab *Scylla serrata* seed in recirculation system. Indones Aquac J 16:107-115. https://doi.org/10.19027/jai.16.1.107-115

Feher LC, Osland MJ, Anderson GH, Vervaeke WC, Krauss KW, Whelan KRT, Balentine KM, Tiling-Range G, Smith TJ, Cahoon DR (2019) The long-term effects of hurricanes Wilma and Irma on soil elevation change in everglades mangrove forests. Ecosystems 23:917-931. https://doi.org/10.1007/s10021-019-00446-x

Franco E, Meleleo C, Serino L, Sorbara D, Zaratti L (2012) Hepatitis A: Epidemiology and prevention in developing countries. World J Hepatol 4:68-73. https://doi.org/10.4254/wjh.v4.i3.68

Go Cheng KJ, Rivera AS, Lam HY, Ulitin AR, Nealon J, Dizon R, Wu DBC (2020) Influenza-associated excess mortality in the Philippines, 2006-2015. PLoS ONE. 15:1-11. https://doi.org/10.1371/journal.pone.0234715

Hallwright J, Handmer J (2019) Accountability and transparency in disaster aid: Cyclone Pam in Vanuatu. Int J Disaster Risk Reduc 36:1-8. https://doi.org/10.1016/j.ijdrr.2019.101104

Hansten M (2017) Harvesting wild Pacific oysters, *Crassostrea gigas*–developing methods for locating and live-storing marketable individuals. Thesis. Yrkeshögskolan Novia

Health Australia (2023) Diarrhoea. Health direct. https://www.healthdirect.gov.au/diarrhoea. Accessed 04 August 2023

Hewitt DR, Duncan PF (2002) Effect of high-water temperature on the survival, moulting and food consumption of *Penaeus (Marsupenaeus) japonicus* (Bate, 1888). Aquac Res 32:305-313. https://doi.org/10.1046/j.1365-2109.2001.00560.x

Hong HK, Park HS, Choi KS (2022) Growth and reproduction of the pacific oyster *Crassostrea gigas* cultured on tidal flat in Hebei spirit oil spill area on the west coast of Korea four years after the accident. Front Mar Sci 9:1-12. https://doi.org/10.3389/fmars.2022.880210

Ji ZL, Li RH, Wang CL, Zhang ZY, Zhang WJ, Mu CK, et al (2022). Effect of long-term low-salinity culture on the survival, growth, and nutrient composition of mud crab *Scylla paramamosain*. J Ocean Univ China 21:179–185. https://doi.org/10.1007/s11802-022-4812-x

Jumawan CQ, Metillo EB, Polistico JP (2021) Assessment of mud crab fishery in Panguil Bay. Philipp J Fish 28:17-32. https://doi.org/10.31398/tpjf/28.1.2020A0002

Kathyayani SA, Muralidhar M, Sathish KT, Alavandi SV (2019) Stress quantification in *Penaeus vannamei* exposed to varying levels of turbidity. In: Jithendran KP, Saraswathy R, Balasubramanian CP, Kumaraguru Vasagam KP, Jayasankar V, Raghavan R, Alavandi SV, Vijayan KK (eds) BRAQCON 2019: World Brackishwater Aquaculture Conference. J Coast Res 86, Florida, pp 177-183

Keriwala N, Patel A (2022) Impact assessment of tropical cyclone Tauktae on coastal region of Gujarat, India. ECS Trans 107:6185–6195. https://doi.org/10.1149/10701.6185ecst

Kishore N, Marques D, Mahmud A, Kiang MV, Rodriquez I, Fuller A, Ebner P, Sorensen C, Racy F, Lemery J, Maas L, Leaning, J, et al. (2018) Mortality in Puerto Rico after Hurricane Maria. N Engl J Med 379:162-170. https://doi.org/10.1056/NEJMsa1803972

Knight KL (2008) More precise classification of orthopaedic injury types and treatment will improve patient care. J Athl Train 43:117-118. https://doi.org/10.4085/1062-6050-43.2.117

Kumar S, Lal P, Kumar A (2021) Influence of super cyclone 'Amphan' in the Indian Subcontinent amid COVID-19 Pandemic. Remote Sens Earth Syst Sci 4:96-103. https://doi.org/10.1007/s41976-021-00048-z

Li D, Huang CY (2018) The influences of ocean on intensity of typhoon Soudelor (2015) as revealed by coupled modeling. Atmos Sci Lett 20:1-7. https://doi.org/10.1002/asl.871

Li C, Zhao Q, Zhao Z, Liu Q, Ma W (2021) The association between tropical cyclones and dengue fever in the Pearl River Delta, China during 2013-2018: A time-stratified case-crossover study. PLoS Negl Trop Dis 15:1-15. https://doi.org/10.1371/journal.pntd.0009776

Liu Z, Lao J, Zhang Y, Liu Y, Zhang J, Wang H, Jiang B (2018) Association between floods and typhoid fever in Yongzhou, China: Effects and vulnerable groups. Environ Res 167:718-724. https://doi.org/10.1016/j.envres.2018.08.030

Long WC, Brylawski BJ, Seitz RD (2008) Behavioral effects of low dissolved oxygen on the bivalve *Macoma balthica*. J Exp Mar Biol Ecol 359:34-39. https://doi.org/10.1016/j.jembe.2008.02.013

Lowe MR, Sehlinger T, Soniat TM, La Peyre MK (2017) Interactive effects of water temperature and salinity on growth and mortality of eastern oysters, *Crassostrea virginica*: A meta-analysis using 40 years of monitoring data. J Shellfish Res 36:663-697. https://doi.org/10.2983/035.036.0318

Lunt J, Smee DL (2014) Turbidity influences trophic interactions in estuaries. Limnol Oceanog 59:2002-2012. https://doi.org/10.4319/lo.2014.59.6.2002

Mangubhai S, Nand Y, Reddy C, Jagadish A (2021) Politics of vulnerability: Impacts of Covid-19 and cyclone Harold on Indo-Fijians engaged in small-scale fisheries. Environ Sci Policy 120:195–203. https://doi.org/10.1016/j.envsci.2021.03.003

Manning EP (2007) Dehydration in extreme temperatures while conducting stability and support operations in a combat zone. Mil Med 172:972-976. https://doi.org/10.7205/MILMED.172.9.972

Marlier ME, Resetar SA, Lachman BE, Anania K, Adams K (2022) Remote sensing for natural disaster recovery: Lessons learned from hurricanes Irma and Maria in Puerto Rico. Environ Sci Policy 132:153–159.

https://doi.org/10.1016/j.envsci.2022.02.023

Mastrangelo G, Fedeli U, Visentin C, Milan G, Fadda E, Spolaore P (2007) Pattern and determinants of hospitalization during heat waves: an ecologic study. BMC Public Health 7:1-8. https://doi.org/10.1186/1471-2458-7-200

McNatt RA, Rice JA (2004) Hypoxia-induced growth rate reduction in two juvenile estuary-dependent fishes. J Exp Mar Biol Ecol 311:147-156. https://doi.org/10.1016/j.jembe.2004.05.006

Monteclaro H, Quinitio G, Moscoso AD, Napata R, Espectato L, Anraku K, Watanabe K, Ishikawa S (2018) Impacts of typhoon Haiyan on Philippine capture fisheries and implications to fisheries management. Ocean Coast Manag 158:128–133. https://doi.org/10.1016/j.ocecoaman.2018.03.032

Mora C, Ospína AF (2001) Tolerance to high temperatures and potentially impact to sea warming on reef fishes of Gorgona Island (tropical eastern Pacific). Mar Biol 139:765–769. https://doi.org/10.1007/s002270100626

Nair A (2021) Tauktae killed 67 people, left 8,629 cattle dead in Gujarat, says Centre. The Indian Express. https://indianexpress.com/article/india/tauktae-killed-67-people-left-8629-cattle-dead-in-gujarat-says-centre-7415713. Accessed 14 March 2023

NHC-NOAA (2014) Hurricane Odile (EP152014). https://www.nhc.noaa.gov/data/tcr/EP152014_Odile.pdf. Accessed 11 March 2023

NHC-NOAA (2018a) Hurricane Michael (AL142018). National Hurricane Center. https://www.nhc.noaa.gov/data/tcr/AL142018_Michael.pdf. Accessed 12 March 2023

NHC-NOAA (2018b) Hurricane Willa (EP242018). National Hurricane Center. https://www.nhc.noaa.gov/data/tcr/EP242018_Willa.pdf. Accessed 12 March 2023

NHC-NOAA (2020) Hurricane Iota (AL213030). National Hurricane Center. https://www.nhc.noaa.gov/data/tcr/AL312020_Iota.pdf. Accessed 12 March 2023

NHC-NOAA (2023) Saffir-Simpson hurricane wind scale. National Hurricane Center and Central Pacific Hurricane Center. National Oceanic and Atmospheric Administration. https://www.nhc.noaa.gov/aboutsshws.php. Accessed 30 August 2023

Nonwachai T, Purivirojku W, Chuchird N, Limsuwan C (2011) Effects of dissolved oxygen levels on growth, survival, and immune response of juvenile pacific white shrimp (*Litopenaeus vannamei)*. Fish Res Bull Kasetsart Univ 35:1-10. https://www.thaiscience.info/journals/Article/KFRB/10892544.pdf

NPR (2018) Hurricane Maria caused 2,975 deaths in Puerto Rico, independent study estimates. https://www.npr.org/2018/08/28/642615337/hurricane-maria-caused-2-975-deaths-in-puerto-rico-independent-study-estimates. Accessed 12 March 2023

Nsereko G, Kadobera D, Okethwangu D, Nguna J, Rutazaana D, Kyabayinze DJ, Opigo J, Ario AR (2020) Malaria outbreak facilitated by appearance of vector-breeding sites after heavy rainfall and inadequate preventive measures: Nwoya District, Northern Uganda, February-May 2018. J Environ Public Health 2020:1-10. https://doi.org/10.1155/2020/5802401

OCHA (2020) Typhoon Vongfong leaves trail of damage as the country grapples with the 'new normal'. United Nations Office for the Coordination of Humanitarian Affairs. https://reports.unocha.org/en/country/asia-

pacific/card/tjXV99QVBE/. Accessed 13 March 2023

Parida BR, Behera SN, Oinam B, Patel NR, Sahoo RN (2018) Investigating the effects of episodic Super-cyclone 1999 and Phailin 2013 on hydro-meteorological parameters and agriculture: An application of remote sensing. Remote Sens Appli: Soc Environ 10:128–137. https://doi.org/10.1016/j.rsase.2018.03.010

Plante S, Chabot D, Dutil JD (1998) Hypoxia tolerance in Atlantic cod. J Fish Biol 53:1342–1356. https://doi.org/10.1111/j.1095-8649.1998.tb00253.x

Rajapakse S (2022) Leptospirosis: clinical aspects. Clin Med (Lond) 22:14-17. https://doi.org/10.7861/clinmed.2021-0784

Reliefweb (2015) Typhoon Soudelor kills 26 in E China. Reliefweb. OCHA Services. https://reliefweb.int/report/china/typhoon-soudelor-kills-26-e-china. Accessed 11 March 2023

Reliefweb (2017) Typhoon Haima emergency appeal final report (MDRPH022). Reliefweb. OCHA Services. https://reliefweb.int/report/philippines/philippines-typhoon-haima-emergency-appeal-final-report-mdrph022. Accessed 11 March 2023

Reliefweb (2019) Tropical cyclone Fani – Apr 2019. Reliefweb. OCHA Services. https://reliefweb.int/disaster/tc-2019-000041-ind. Accessed 11 March 2023

Reliefweb (2023) The Bahamas: Hurricane Dorian – Final Report (MDRBS003). Reliefweb. OCHA Services. https://reliefweb.int/report/bahamas/bahamas-hurricane-dorian-final-report-mdrbs003. Accessed 12 March 2023

Roberts SD, Van Ruth PD, Wilkinson C, Bastianello SS, Bansemer MS (2019) Marine heat wave, harmful algae blooms and an extensive fish kill event during 2013 in South Australia. Front Mar Sci 6:1-20. https://doi.org/10.3389/fmars.2019.00610

Rosas C, Martinez E, Gaxiola G, Brito R, Sanchez A, Soto LA (1998) The effect of dissolved oxygen and salinity on oxygen consumption, ammonia excretion and osmotic pressure of *Penaeus setiferus* (Linnaeus) juveniles. J Exp Mar Biol Ecol 234:41-57. https://doi.org/10.1016/S0022-0981(98)00139-7

Ruscoe IM, Shelley CC, Williams GR (2004) The combined effects of temperature and salinity on growth and survival of juvenile mud crabs (*Scylla serrata* Forskål). Aquac 238: 239-247. https://doi.org/10.1016/j.aquaculture.2004.05.030

Sanda T, Shimizu T, Dan S, Hamasaki K (2022) Temperature tolerance of juveniles of two mud crab species, *Scylla paramamosain* and *Scylla serrata* (Decapoda: Brachyura: Portunidae). Crust Res 51:129-135. https://doi.org/10.18353/crustacea.51.0_129

Sanders JA, Warren B, Hanrahan T, Wright W (2015) Sand Wars. Excessive sedimentation on Grays Harbor oyster beds: Likely causes and recommended mitigation. Grays Harbor Conservation District. https://www.ezview.wa.gov/Portals/_1492/Documents/01-Final%20main%20report%20on%20sediment%20mitigation%20for%20GH%20oyster%20beds%2006-30-15.pdf. Accessed 20 July 2023

Santos CT, Toda L, Orduna JR, Santos FD, Derrao J (2016) The impacts of typhoon Haiyan in the Philippines: Implications to land use planning. Clim Disaster Dev J 1:57-66. https://doi.org/10.18783/cddj.v001.i01.a06

Santos GDC (2021) 2020 tropical cyclones in the Philippines: A review. Trop Cyclone Res Rev 10:191–199. https://doi.org/10.1016/j.tcrr.2021.09.003

Seager J, Milne I, Mallett M, Sims I (2000) Effects of short-term oxygen depletion on fish. Environ Toxicol Chem 19:2937-2942. https://doi.org/10.1002/etc.5620191214

Sehlinger T (2018) Analysis of temperature and salinity effects on growth and mortality of oysters (*Crassostrea virginica*) in Louisiana (2018). Dissertation, University of New Orleans. https://scholarworks.uno.edu/td/2515

Senkbeil JC, Myers L, Jasko S, Reed JR, Mueller R (2020) Communication and hazard perception lessons from category five hurricane Michael. Atmosphere 11:1-19. https://doi.org/10.3390/atmos11080804

Shives JA, Dunbar SG (2010) Behavioral responses to burial in the hermit crab, *Pagurus samuelis*: Implications for the fossil record. J Exp Mar Biol Ecol 388:33-38. https://doi.org/10.1016/j.jembe.2010.03.008

UNESCAP (2021) Cyclone Tauktae: a perfect storm of climate change and pandemic. ICT and disaster risk reduction. United Nations Economic and Social Commission for Asia and Pacific. https://www.unescap.org/blog/cyclone-tauktae-perfect-storm-climate-change-and-pandemic. Accessed 15 June 2023

van Vuuren DP, Edmonds J, Kainuma M, Riahi K, Thomson A, Hibbard K, Hurtt GC, Kram T, Krey V, Lamarque J-F, Masui T, Meinshausen M, Nakicenovic N, Smith SJ, Rose SK (2011) The representative concentration pathways: an overview. Clim Change 109:5-31. https://doi.org/10.1007/s10584-011-0148-z

Ventura RJ, Muhi E, delos Reyes VC, Sucaldito MN, Tayag E (2015) A community-based gastroenteritis outbreak after Typhoon Haiyan, Leyte, Philippines, 2013. Western Pac Surveill Response J 6:1–6. https://doi.org/10.2471/WPSAR.2014.5.1.010

Vinagre C, Leal I, Mendonca V, Flores AAV (2015) Effect of warming rate on the critical thermal maxima of crabs, shrimp and fish. J Therm Biol 47:19-25. https://doi.org/10.1016/j.jtherbio.2014.10.012

Wang T, Li Q (2018) Effects of salinity and temperature on growth and survival of juvenile Iwagaki oyster *Crassostrea nippona.* J Ocean Univ China 17:941-946. https://doi.org/10.1007/s11802-018-3536-4

Whitfield AK, Taylor RH, Fox C, Cyrus DP (2006) Fishes and salinities in the St Lucia estuarine system—a review. Rev Fish Biol Fish 16:1-20. https://doi.org/10.1007/s11160-006-0003-x

WHO (2010) The global prevalence of Hepatitis A virus infection and susceptibility: A systematic review. World Health Organization, Geneva

WHO (2022) Cholera-global situation. World Health Organization. https://www.who.int/emergencies/disease-outbreak-news/item/2022-DON426. Accessed 05 August 2023

Wingard GL, Bergstresser SE, Stackhouse BL, Jones MC, Marot ME, Hoefke K, Daniels A, Keller K (2019) Impacts of Hurricane Irma on Florida Bay Islands, Everglades National Park, USA. Estuar Coast 43:1070–1089. https://doi.org/10.1007/s12237-019-00638-7

World Bank (2014) India averts devastation from cyclone Phailin. World Bank. https://www.worldbank.org/en/results/2014/04/10/india-averts-cyclone-phailin-devastation. Accessed 12 March 2023

World Bank (2015) Vanuatu: Six months after cyclone Pam. World Bank.

https://www.worldbank.org/en/news/feature/2015/09/29/vanuatu-six-months-after-cyclone-pam. Accessed 12 March 2023

World Vision (2020) 2017 Hurricane Irma: Facts, FAQs, and how to help. World Vision. https://www.worldvision.org/disaster-relief-news-stories/2017-hurricane-irma-facts. Accessed 12 March 2023

Yin S, Lin X, Yang S (2022) Characteristics of rainstorm in Fujian induced by typhoon passing through Taiwan Island. Trop Cyclone Res Rev 11:50-59. https://doi.org/10.1016/j.tcrr.2022.04.003

Young BC, Alfaggeh RH, AlMoutri I, Cheng W (2022) Effects of high salinity on survival, growth, and reproductive performance of red tilapia (*Oreochromis urolepis hornorum* ♀× *Oreochromis mossambicus* ♂). Isr J Aquac - Bamidgeh IJA.74:1-5. https://doi.org/10.46989/001c.32371

Zhang P, Li J, Huang G, Zhang X (2006) The effects of body weight, temperature, salinity, pH, light intensity and feeding condition on lethal DO levels of whiteleg shrimp, *Litopenaeus vannamei* (Boone, 1931). Aquac 256: 579-587. https://doi.org/10.1016/j.aquaculture.2006.02.020

zu Ermgassen PSE, Mukherjee N, Worthington TA, Acosta A, Rocha Araujo AR, da Beitl CM, Castellanos-Galindo GA, Cunha-Lignon M, Dahdouh-Guebas F, Diele K, Parrett CL, Dwyer PG, Gair JR, Johnson AF, Kuguru B, Savio Lobo A, Loneragan NR, Longley-Wood K, Mendonça JT, Meynecke JO (2020) Fishers who rely on mangroves: Modelling and mapping the global intensity of mangrove-associated fisheries. Estuar Coast Shelf Sci 247:1-10. https://doi.org/10.1016/j.ecss.2020.106975
